# Transcriptome Analysis of Oleoresin-Producing Tree *Sindora Glabra* and Characterization of Sesquiterpene Synthases

**DOI:** 10.3389/fpls.2018.01619

**Published:** 2018-11-20

**Authors:** Niu Yu, Jin-Chang Yang, Guang-Tian Yin, Rong-Sheng Li, Wen-Tao Zou

**Affiliations:** Key Laboratory of State Forestry Administration on Tropical Forestry Research, Research Institute of Tropical Forestry, Chinese Academy of Forestry, Guangzhou, China

**Keywords:** transcriptome, *Sindora glabra*, oleoresin, terpene biosynthesis, terpene synthase

## Abstract

Terpenes serve important physiological and ecological functions in plants. *Sindora glabra* trees accumulate copious amounts of sesquiterpene-rich oleoresin in the stem. A transcriptome approach was used to determine the unique terpene biosynthesis pathway and to explore the different regulatory mechanisms responsible for the variation of terpene content among individuals. Analysis of *de novo-*assembled contigs revealed a complete set of genes for terpene biosynthesis. A total of 23,261 differentially expressed unigenes (DEGs) were discovered between high and low oil-yielding plants. DEG enrichment analysis suggested that the terpene biosynthesis process and the plant hormone signal transduction pathway may exert a major role in determining terpene variation in *S. glabra*. The expression patterns of candidate genes were further verified by quantitative RT-PCR experiments. Key genes involved in the terpene biosynthesis pathway were predominantly expressed in phloem and root tissues. Phylogenetic analysis and subcellular localization implied that *S. glabra* terpene synthases may evolve from a common ancestor. Furthermore, two sesquiterpene synthase genes, *SgSTPS1* and *SgSTPS2*, were functionally characterized. SgSTPS1 mainly generated β-caryophyllene from farnesyl pyrophosphate. SgSTPS2 is a versatile enzyme that catalyzes the formation of 12 sequiterpenes from farnesyl pyrophosphate and synthesis of three monoterpenes using geranyl pyrophosphate. Together, these results provide large reservoir for elucidating the molecular mechanism of terpene biosynthesis and for exploring the ecological function of sesquiterpenes in *S. glabra*.

## Introduction

The angiosperm Caesalpinioideae subfamily includes the important genera *Copaifera* Linn., *Hymenaea* Linn., and *Sindora* Miq., which are traditionally referred to as “diesel trees” by local people (Langenheim, 2003). These diesel trees produce a sesquiterpene-rich oleoresin that is routinely collected when the plant trunk is drilled into and tapped.Oleoresin has been widely utilized in pharmaceuticals, fuel, essential oils and food (Peltier et al., 2006; Gershenzon and Dudareva, 2007; Harvey et al., 2010). However, the focus of oleoresin production research in plants has been predominantly limited to the genus *Copaifera*, which is distributed in tropical America and Africa (Souza Barbosa et al., 2013; Amorim et al., 2017). The genus *Sindora*, naturally distributed in the tropical forests of Asia and Africa, contains the species *Sindora glabra*, which is indigenous to Hainan Island, China. The *S. glabra* tree exudes yellowish oleoresin or an amber liquid oil when wounded that has been conventionally used as kerosene. Due to its endangered state this species has been included in the second-class national protection plants of China. Studies have shown that the annual oleoresin yield of *S. glabra* varies from 0.01 to 3.00 L among different individuals and the major components of the resin oil are sesquiterpenes (about 85%) and abietic acid (about 13%) (Yang et al., 2016), which is a unique feature of *S. glabra* oleoresin. Variation in the oil composition is present across the natural distribution range of *S. glabra*.

Terpenes are one of the largest and most diverse class of plant metabolites and serve essential functions in plant growth, development, and defense. Moreover, many terpenes of specialized metabolites, such as artemisinin from *Artemisia annua* and paclitaxel from *Taxus baccata*, have high medical value (Klayman, 1985; Sandler et al., 2006). However, the amount and composition of terpenes differ greatly among plant species and tissues. The gymnosperm *Pinus* stores oleoresin in specialized resin canals and produces higher yields in spring (0.65 kg) and summer (0.55 kg) compared to autumn and winter (Lombardero et al., 2000; Rodrigues and Fett-Neto, 2009). Moreover, gymnosperm oleoresin is almost universally composed of mono- and di-terpenes. Paclitaxel are mainly extracted from tree bark, while tanshinone from *Saliva miltiorrhiza* is extracted from roots (Ge and Wu, 2005). The underlying molecular mechanisms of terpene biosynthesis have been thoroughly studied (Tholl, 2006; Degenhardt et al., 2009). Terpenes are polymers of isoprene and are derived from the five carbon units of isopentyl diphosphate (IPP) and dimethylallyl diphosphate (DMAPP), which are generated from the plastidic methylerythritol phosphate (MEP) pathway or the cytoplasmic mevalonate (MVA) pathway. Condensation of IPP and DMAPP by prenyltransferase contribute to the three linear intermediates, geranyl diphosphate (GPP), farnesyl diphosphate (FPP), and geranylgeranyl diphosphate (GGPP), which are then catalyzed by terpene synthases (TPSs) to form monoterpenes (C_10_), sesquiterpenes (C_15_), and diterpenes (C_20_) (Lichtenthaler, 1999). Generally, the MEP pathway generates monoterpenes and diterpenes, whereas the MVA pathway produces sesquiterpenes and triterpenes. There is also some cross-talk between these two pathways, for example, the non-MVA pathway synthesizes both monoterpenes and sesquiterpenes in roots and leaves of *Daucus carota* (Hampel et al., 2005). Products of various TPSs are further subject to structural modification through oxidation, reduction, isomerization, hydration, and conjugation to give rise to the chemical diversity of terpenes (McGarvey and Croteau, 1995). Many TPSs can utilize the same substrate to produce multiple products and the variation of even a single amino acid mutation in conserved domains of TPSs can alter product profiles (Li J. X. et al., 2013). Plant genomes typically contain families of many TPSs with similar sequences but that are functionally diverse, with gene numbers ranging from ~20 to 150 (Chen et al., 2011). Variation in the genome and expression levels of TPSs may explain some of the variation in terpenes present in natural *S. glabra* populations. Substantial progress has been achieved in the discovery and elucidation of TPSs involved in terpene biosynthesis in tree species, including the gymnosperms *Abies grandis, Picea abies*, and *Ginkgo biloba*, and the angiosperms *Melaleuca alternifolia, Santalum spicatum*, and *Populus trichocarpa* (Warren et al., 2015; Bustos-Segura et al., 2017).

Factors affecting terpene biosynthesis are quite complex and could include the plant developmental stage, biotic factors, such as insects and pathogens, and abiotic factors, such as light, temperature, and humidity. In *Picea* spp., stem resin accumulates constitutively in the cortex, while it appears within the developing xylem after mechanical wounding, insect feeding, or fungal elicitation (Martin et al., 2002). Some transcription factors, including the MYB, ERF, YABBY, and NAC families, have been found to regulate the biosynthesis of terpene secondary metabolites (Nieuwenhuizen et al., 2015; Wang et al., 2016; Li et al., 2017; Matías-Hernández et al., 2017). Plant signaling molecules, especially jasmonic acid (JA), have great potential to elicit the production of terpenes in gymnosperms, herbs and crops (Martin et al., 2003; Kim et al., 2006; Ghasemzadeh et al., 2016). Herbivore-induced diterpene resin in conifer trees were discovered to serve important defense functions (Keeling and Bohlmann, 2006; Hall et al., 2013). In *Sitka spruce*, traumatic resin and terpene synthase transcripts were induced following attack by white pine weevils, and the defense response was more complex than that associated with methyl jasmonate (Miller et al., 2005).

Elucidation of terpene biosynthesis in *S. glabra* provides a basis to understand oil composition and variability. With a deeper understanding of the genetic mechanism of metabolic pathways in this species, we could improve our understanding of the developmental and physiological conditions that are responsible for the production of the resin oil. This in turn may support breeding efforts toward tree improvement for oil yield and quality in sustainable *S. glabra* plantations and can afford opportunities for biotechnological *S. glabra* oil production (Bustos-Segura et al., 2017). Some important genes can be identified for enhanced production of a particular terpene in either microorganisms or other plant species. In this study, comprehensive *de novo* transcriptome analysis of high and low oil-yielding *S. glabra* was carried out. Key genes related to terpene biosynthesis were mined, and the expression patterns were validated in experiments. The function of *S. glabra* sesquiterpene synthase was further characterized.

## Materials and methods

### Plant materials

Plants were grown from seeds from eight natural populations of *S. glabra* distributed in Hainan Island in glasshouse with 16 h light photoperiods. After 1 year, seedlings were transferred to plantations located in the Experimental Center of Tropical Forestry, Chinese Academy of Forestry in Pingxiang City, Guangxi, China (22°02′-22°19′N, 106°43′-106°52′E). Plants were maintained for 10 years before sampled. We selected two plants L4 and L6 (annual yield of 25 and 50 g) as low oil-yielding plants and two plants H10 and H12 (annual yield of 475 and 770 g) as high oil-yielding plants. Each tissue was collected from three individuals in the same family representing biological replicates. Fresh stem tissues were sampled after peeling and immediately frozen in liquid nitrogen and stored at −80°C for RNA extraction.

### Structure analysis and determination of terpene profile

Samples were prepared for cryosectioning as described by Martin et al. (2002). Sections were stained with 1% carmine and astra blue. Final sections were placed on the glass lamina and photographed with a light microscope Olympus BH-2. Extraction of terpene constituents were done as Lewinsohn et al. (1993). The extract was used for GC-MS analysis (Agilent 6890). Chemical compounds were identified based on comparison of the mass spectra with National Institute of Standards and Technology (NIST) standard library. Relative percentage of the identified compounds were computed based on the peak area.

### RNA library construction and sequencing

Total RNA was extracted from each sample using cetyltrimethyl ammonium bromide (CTAB) method as described by Asif et al. (2000), plus the Plant RNA isolation kit. Briefly, fine powder were extracted with the buffer containing 2% CTAB and chloroform was added to remove polyphenols and polysaccarides. The aqueous phase were then subject to RNA isolation according to manufacturer's protocol (Qiagen RNeasy kit). RNA concentration was measured using Qubit RNA assay kit in Qubit 2.0 Flurometer (Life Technologies, USA). RNA integrity was assessed by using the Agilent Bioanalyzer 2100 system (Agilent Technologies, USA). A total of 1.5 μg RNA per sample was used as input material for the RNA sample preparations. Sequencing libraries were generated using NEBNext Ultra RNA Library Prep Kit for Illumina (NEB, USA) following manufacturer's recommendations. The total of 12 libraries were subject to sequencing on an Illumina Hiseq 2000 platform and paired-end reads were generated.

### *De novo* transcriptome assembly

Clean reads were obtained by removing reads containing adapter, poly-N and low quality reads. The high-quality reads were then *de novo* assembled using the Trinity platform (https://github.com/trinityrnaseq/trinityrnaseq/wiki) with the parameters “K-mer=25, min_kmer_cov=2.” The reads obtained for all samples were assembled together. Short reads were first assembled into draft transcript contigs, then pooled into components and finally assembled into transcripts. The longest transcript was taken as one unigene. All transcriptome sequence data were deposited in NCBI Short Read Achieve (SRA) database under the accession number SRP133897.

### Gene functional annotation

Gene putative function was annotated by using diamond v0.8.22 against the NCBI non-redundant protein sequences (Nr) database (e-value = Ie^−5^), NCBI blast against the NCBI non-redundant nucleotide sequences (Nt) database (e-value = Ie^−5^), HMMER 3.0 against the Protein family (Pfam) database (e-value = 0.01), diamond v0.8.22 against the Cluster of Orthologous Groups of proteins (KOG/COG) database (e-value = Ie^−3^), diamond v0.8.22 against A manually annotated and reviewed protein sequence database (Swiss-Prot) database (e-value = Ie^−5^), KEGG automatic annotation server against the Kyoto Encyclopedia of Genes and Genomes (KEGG) database (e-value = Ie^−10^), and Blast2GO against the Gene Onthology (GO) database (e-value = Ie^−6^). Transcription factors (TFs) were identified by doing BLASTx against all plant transcription factors database (http://planttfdb.cbi.pku.edu.cn/), at e-value 1e^−5^ and query coverage 50%. The CDS prediction was performed by blast into nr and Swissprot protein database and the ORF protein coding sequences were extracted and translated into protein sequences according to standard codon. For those unmapped sequences or mapped but with no predicted sequences the sequences were subjected to prediction by estscan (3.0.3) software.

### Gene expression analysis

Gene expression levels were estimated by RSEM for each sample. The clean data were mapped back onto the assembled transcriptome. Readcount for each gene was obtained from the mapping results and normalized into a FPKM value (expected number of Fragments Per Kilobase of transcript sequence per Millions base pairs sequenced, Trapnell et al., 2010). Differential expression analysis of two conditions was performed using the DESeq R package. The resulting *P*-values were adjusted using the Benjamini and Hochberg's approach for controlling the false discovery rate. Unigenes with an adjusted *P*-value (*p*adj) < 0.05 found by DESeq were assigned as differentially expressed. Gene Ontology (GO) enrichment analysis of the differentially expressed unigenes (DEGs) was implemented by the GOseq R packages based Wallenius non-central hyper-geometric distribution, which can adjust for gene length bias in DEGs. The KOBAS software was used to test the statistical enrichment of DEGs in KEGG pathways. Gene expression correlation network between candidate STPS genes and WRKY genes was constructed with the Weighted Gene Co-expression Network Analysis (WGCNA) method (Langfelder and Horvath, 2008). Heat map was produced by the software Heatmap 2.0 (Toddenroth et al., 2014).

### Quantitative RT-PCR analysis

For qRT-PCR validation, all RNA samples were reverse transcribed into first-strand cDNA using Superscript III 1st strand RT-PCR reactions according to the manufacturer's protocol (Invitrogen, USA). Amplifications were carried out in triplicate in a total volume of 20 μL using SYBR Premix Ex Taq™ II kit (Takara). The specificity of the PCR amplicon was checked using a heat dissociation protocol (from 60 to 95°C) after the final PCR cycle. Three independent biological replicates and three technical replicates were performed. The primers used in the qPCR were shown in File S12 and the *ACT2* gene was used as internal control to normalize the expression.

### Sequence analysis of SgTPSs

Prediction of the presence of putative signal peptide was carried out using SignalIP 4.1 program (http://www.cbs.dtu.dk/services/SignalP/). The homology search was performed using BLAST server. The multiple alignment of amino acid sequences were accomplished using the ClustalW2 program (http://www.ebi.ac.uk/Tools/msa/clustalo/). For phylogenetic analysis, the *Arabidopsis thaliana* and *Populus trichocarpa* terpene synthase sequences were retrieved from TAIR database (https://www.arabidopsis.org/) and *Populus trichocarpa* Genome database (http://www.plantgdb.org/PtGDB/). The bootstrapped neighbor joining tree was constructed using the MEGA 7 program (Tamura et al., 2011).

### Subcellular localization of SgTPSs

Leaves of 4 weeks old *Nicotiana benthamiana* plants were used for agrobacterium-mediated infiltration as described by Wydro et al. (2006). The ORF sequence of each gene was cloned into pCambia1301 vector and the GFP fusion constructs were used for transient expression. Infiltrated leaves were mounted on slides and imaged using a confocal laser-scanning microscope (Nikon C2-ER) with a standard filter set. The empty GFP vector was used as control.

### Preparation of recombinant proteins

The identified terpene synthase gene was cloned from *S. glabra* cDNA library and confirmed by sequencing. The gene was then subcloned into pET30a vector for prokaryote expression. The resulting expression constructs were confirmed by restriction digestion and sequencing. For functional expression, the constructs were transformed into BL21 (DE3) cells. Cultures containing the recombinant constructs were grown overnight at 37°C in LB medium with antibiotics and inoculated into LB medium until OD600 reached to 0.8, then induced with 0.1 mM isopropyl β-D-1-thiogalactopyranoside (IPTG) for 16 h at 15°C. The induced cell pellets were collected for cell lysis and the supernatants containing soluble target proteins were used for protein purification by affinity chromatography on nickel-iminodiacetic acid (Ni-IDA) resin (Qiagen, Germany). Protein concentration was determined using the Bradford method.

### *In vitro* assay and GC-MS analysis

For *in vitro* assay, the reactions were carried out in 500 μL assay buffer (25 mM Tris-HCl PH 7.0, 5 mM DTT, 5 mM MgCl_2_) containing 5 μg purified protein and 50 μM GPP or FPP substrate. The contents were mixed in a 2 ml glass vial and incubated at 30°C for 5 h. Then the reaction products were extracted with solid-phase microextraction (SPME) system for 30 min and analyzed by gas chromatography-mass spectrometry (GC-MS) system 7890B-5977A (Agilent Technologies) fitted with HP-5MS column (30 m × 0.25 mm). The injection temperature was 250°C with ionization energy 70 eV and mass scan range 30–300 amu. The GC was programmed with an initial temperature of 50°C for 1 min, then increasing at a rate 5°C/min until 80°C (1 min hold), and then at a rate of 10°C/min until 220°C (10 min hold). Identification of compounds were achieved by using the NIST14 mass spectral library database and by comparison of retention times and mass spectra with authentic standards if available.

## Results

### GC-MS profile of oleoresin in *S. glabra*

**Figure 1:**
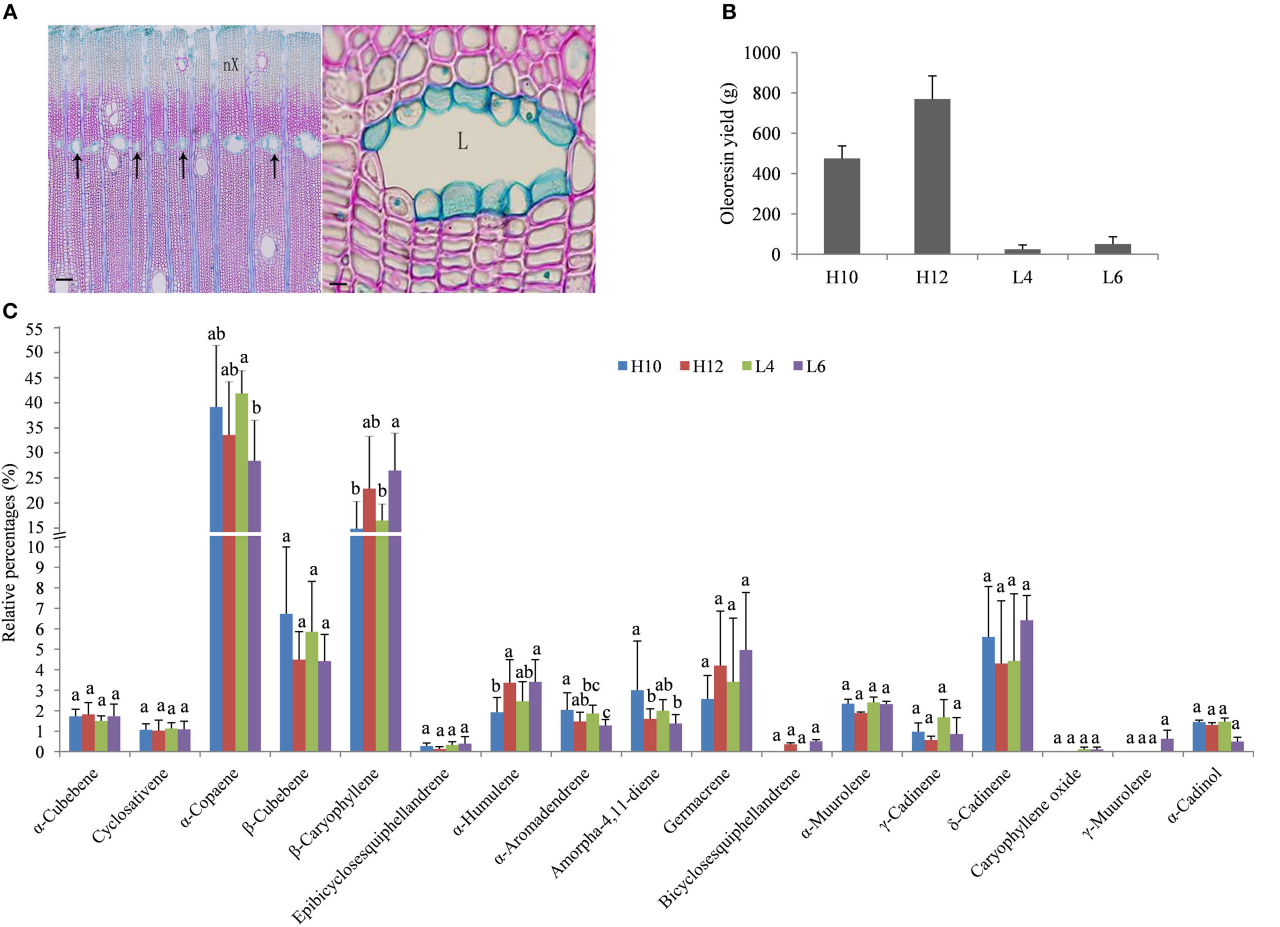
Structure of secretory canals and terpene profile in the *S. glabra* stem. **(A)** Anatomical structure of secretory canals. Left, secretory canals distributed along the marginal parenchyma bands (arrow); Right, secretory canals at the mature stage; nX, New xylem; L, Lumen; Scale bars: Left, 100 μm; Right, 10 μm. **(B)** Oleoresin yield in *S. glabra* plants. **(C)** Chemical composition of terpenes by GC-MS. Error bars indicate the standard error. The different lower case letters above the columns indicate significant differences based on ANOVA (*p* < 0.05).

The trunk of *S. glabra* can exude a large amount of oleoresin when drilled into or tapped. These oleoresin are stored in specific cells named secretory canals. Anatomical analysis of *S. glabra* stem tissue showed secretory canals exist in the secondary xylem of the trunk (Figure 1A). To gain more insight into the molecular mechanism of oleoresin biosynthesis and excretion in *S. glabra*, two high oil-yielding and two low oil-yielding plants were selected for oleoresin analysis (Figure 1B). The annual oleoresin yield in H12 plants was ~30 times higher than that of L4 plants. The GC-MS profile showed that the chemical compounds of oleoresin in the four types of plants were very similar (Figure 1C). There were about 18 types of sesquiterpenes that had identifiable levels of >0.1% of total compounds detected. The major sesquiterpenes were α-copaene and β-caryophyllene, followed by β-cubebene, δ-cadinene, and germacrene. The other 13 compounds accounted for <3%. The relative amount of five compounds, namely, α-copaene, β-caryophyllene, α-humulene, aromadendrene, and amorpha-4,11-diene, showed a statistical difference among the four samples, while the other compounds were not statistically diverse.

### RNA-Seq and *de novo* transcriptome assembly

The stem tissue of *S. glabra* was used for RNA-seq library construction. Transcriptome sequencing of 12 cDNA libraries generated an average of 55.51 million raw data reads (Table S1). After filtering adapter, low quality, and short reads, an average of 9.2 G clean bases were obtained. The Q30 percentages (percentage of bases with phred value >30) were above 93%, and the GC content was about 43.63%. Furthermore, the valid reads were *de novo* assembled to generate 409,106 transcripts, which were further clustered into 283,998 unigenes (Table S2). The mean unigene length was 1,701 bp, and the N50 length was 2,541 bp. The largest number of unigenes was found for a size >2,000 bp, followed by unigenes ranging from 1,000 to 2,000 bp, and then unigenes between 500 and 1,000 bp. These data indicated high accuracy and good assembly of this transcriptome sequencing.

### Functional annotation

All of the unigenes were searched against seven public databases for functional annotation and there were 231,334 unigenes annotated in at least one database and 38,014 unigenes annotated in all of the databases (Figure S1A, Table S3). The annotation percentage was highest in the nr database and lowest in the KOG database. The Venn map revealed that 48,447 unigenes were found to be common among five databases.

Based on nr annotation and the *E*-value distribution, 75.59% of unigenes showed homology to annotated genes from 818 plant species (Figure S1B, File S1). Among these, the top five species were *Glycine max, Glycine soja, Cicer arietinum, Phaseolus vulgaris*, and *Medicago truncatula*, all of which belong to the Fabaceae family. GO annotation revealed that in total, 154,112 unigenes were classified into three GO categories (Figure S1C, File S2). In the biological process category, large numbers of unigenes were classified into the cellular process and metabolic process groups, including 102 and 5,744 unigenes related to the fatty acid derivative metabolic process and the lipid metabolic process, respectively, which implied the identification of genes involved in specific metabolite biosynthesis pathways. In the molecular function category, genes related to binding and catalytic activity were highly abundant, indicating large amounts of enzymes in this particular state.

All of the unigenes were subjected to a search against the KOG database, resulting in the assignment of 63,438 unigenes (Figure S1D, File S3). These unigenes were classified into 26 different functional groups, with the largest cluster being groups O and R, followed by group J. It was noteworthy that there were 2,986 and 957 unigenes annotated in the lipid transport and metabolism group (group I) and the secondary metabolites biosynthesis group (group Q), respectively.

According to mapping in reference to the canonical pathways in KEGG, a total of 90,343 unigenes were assigned to 130 pathways (Figure S1E, File S4). Among these, carbon metabolism was the most enriched, followed by the biosynthesis of amino acids. In addition, there were 1,838 unigenes involved in plant hormone signal transduction. These genes can be good candidates for investigating plant growth regulation and stress response. Moreover, there were 2,131 unigenes involved in the metabolism of terpenoids and polyketide pathways. This information is particularly important for the identification of genes involved in terpenoid biosynthesis in *S. glabra*.

### Analysis of differentially expressed unigenes

**Figure 2:**
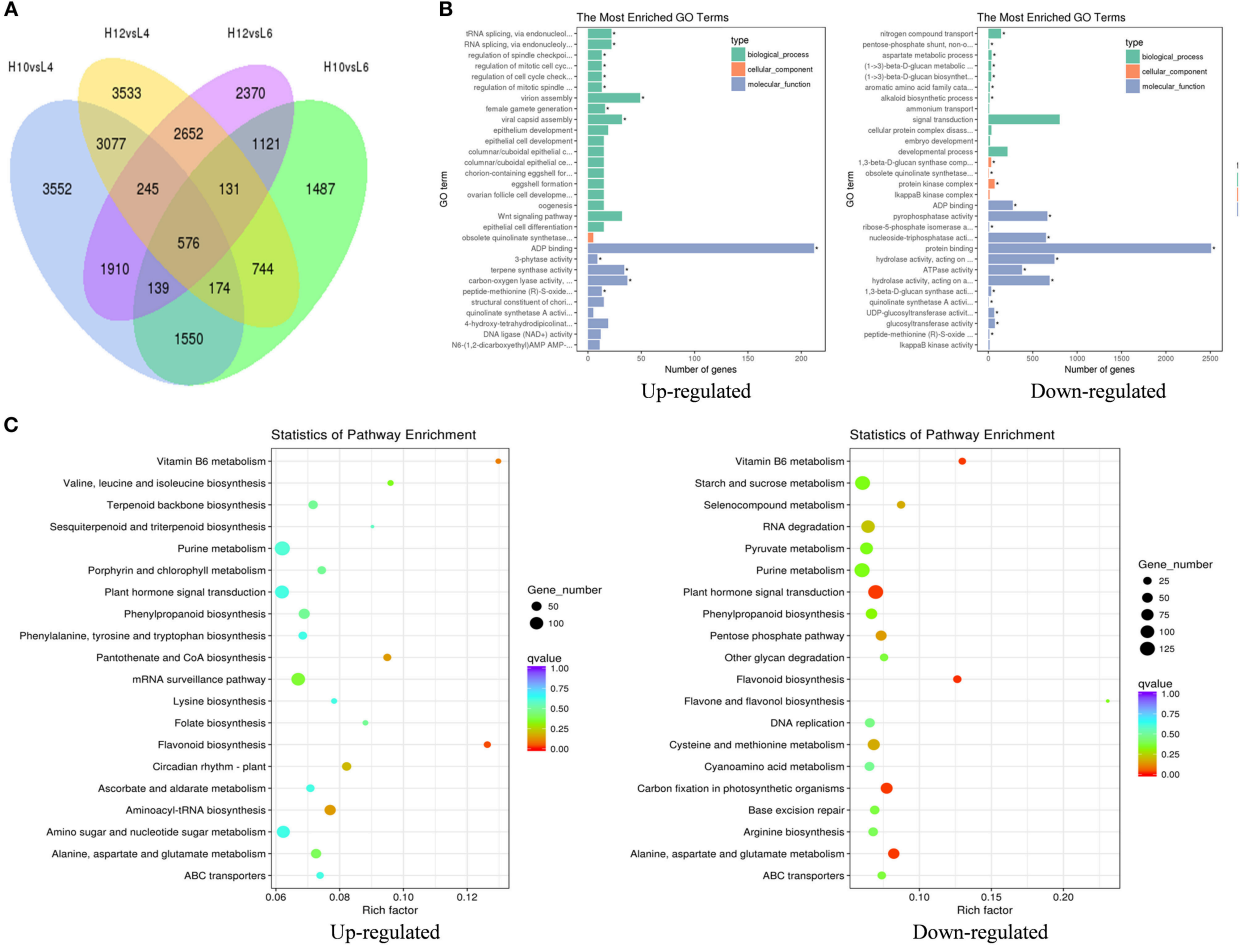
Identification and classification of differentially expressed genes (DEGs) among samples. **(A)** Venn diagram of DEGs among four pairs of samples. **(B)** GO enrichment analysis of up- and down-regulated DEGs in H10 and H12 relative to L4 and L6. The top 30 enriched GO terms are shown. The asterisks indicate significantly enriched terms (*P*adj < 0.05). **(C)** KEGG pathway enrichment analysis of up- and down-regulated DEGs in H10 and H12 relative to L4 and L6. The top 20 enriched KEGG pathways are shown.

The transcripts assembled by Trinity were used as the reference transcriptome, and the clean reads from each sample were mapped to the reference sequences. The average mapping rate was 80.85%. The differentially expressed genes (DEGs) were analyzed by comparing high oil-yielding plants with low oil-yielding plants: pair H10 and L4, H10 and L6, H12 and L4, and H12 and L6, respectively. There were 9,797 unigenes up-regulated in H10 and H12, and 9,393 unigenes were predominantly expressed in L4 and L6 (Figure 2A). The greatest gene expression difference existed between H10 and L4, and between H12 and L4, followed by pair H12 and L6, and pair H10 and L6. It should be noted that there were 10, 9, 15, and 11 down-regulated and 40, 21, 3, and 14 up-regulated DEGs annotated in the terpenoid biosynthesis pathway between pairs H10 and L4, H12 and L4, H12 and L6, and H10 and L6, respectively (File S5). These DEGs involved in the terpenoid pathway could serve as good candidates for functional validation. Moreover, when comparing the expression profiles of H10 and H12 to those of L4 and L6, a total of 273 unigenes were co-repressed, and 248 unigenes were co-induced, respectively (File S6). These DEGs were involved in signaling, protein metabolism and processing, defense, transcription factors, and metabolism, suggesting that signaling pathways, transcription factors, and metabolism may be responsible for *S. glabra* terpene polymorphism.

Based on the GO functional enrichment, the up-regulated DEGs in H10 and H12 were significantly enriched in 14 GO terms (*P*adj < 0.05, Figure 2B), among which the most enriched were related to ADP binding in the molecular function category. In particular, there were 34 DEGs significantly enriched in terpene synthase activity, which implied positive roles of these DEGs in the regulation of terpene synthesis. When analyzing the down-regulated DEGs in H10 and H12, there were 803 DEGs enriched in signal transduction in the biological process, suggesting possible negative regulation of these DEGs in oil yield.

Furthermore, KEGG pathway enrichment of these DEGs resulted in the assignment of 121 metabolic pathways. The flavonoid biosynthesis pathway was significantly enriched in H10 and H12 predominantly expressed DEGs (Figure 2C). In addition, there were 43 and 13 up-regulated DEGs enriched in terpenoid backbone biosynthesis and the sesquiterpenoid and triterpenoid biosynthesis pathway, respectively, which were consistent with the GO enrichment results that terpene biosynthesis-related DEGs could increase the terpene content. In L4 and L6 predominantly expressed DEGs, five KEGG pathways were significantly enriched, among which the majority of DEGs were involved in the plant hormone signal transduction pathway, further implying the potential influence of plant signal transduction-related DEGs in the down-regulation of terpene yield. Together, these data suggested that the terpene biosynthesis process and plant hormone signal transduction pathway may exert the most significant role in determining the terpene variation in *S. glabra*.

### Terpene biosynthesis in *S. glabra*

**Figure 3:**
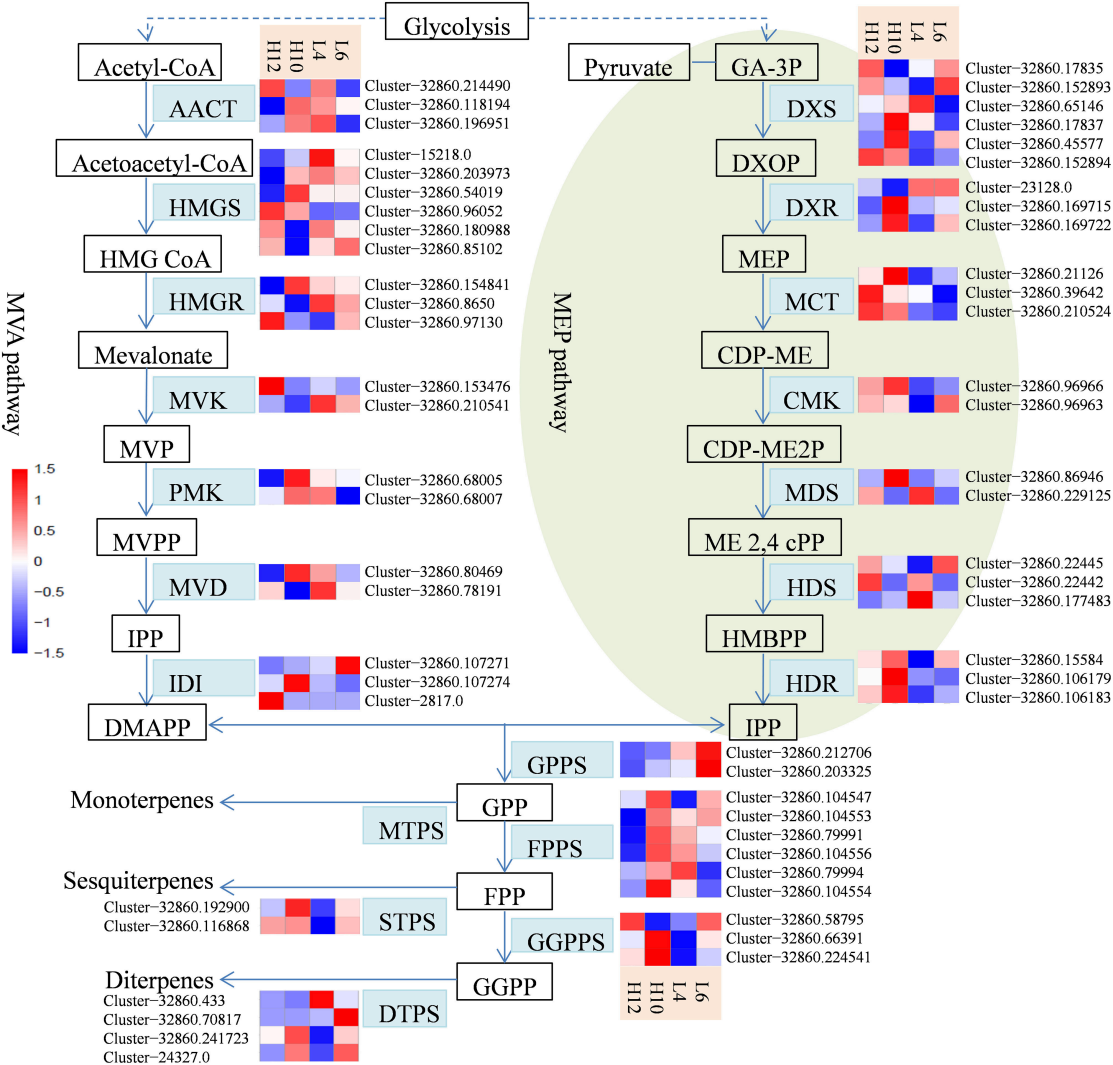
Heat maps showing the different expression profiles of genes involved in the terpene biosynthesis pathway. The scale represents log2 fold changes in H10 and H12 relative to L4 and L6. Unigenes with an adjusted *P*-value (*p*adj) < 0.05 were differentially expressed. AACT, acetyl-CoA acetyltransferase; HMGS, hydroxymethylglutaryl-CoA synthase; HMGR, hydroxymethylglutaryl-CoA reductase; MVK, mevalonate kinase; PMK, phosphomevalonate kinase; MVD, mevalonate diphosphate decarboxylase; DXS, 1-deoxy-D-xylulose 5-phosphate synthase; DXR, 1-deoxy-D-xylulose-5-phosphate reductoisomerase; MCT, 2-C-methyl-D-erythritol 4-phosphate cytidylyltransferase; CMK, 4-diphosphocytidyl-2-C-methyl-D-erythritol kinase; MDS, 2-C-methyl-D-erythritol 2,4-cyclodiphosphate synthase; HDS, (E)-4-hydroxy-3-methylbut-2-enyl-diphosphate synthase; HDR, 4-hydroxy-3-methylbut-2-enyl diphosphate reductase; IDI, isopentenyl di-phosphate isomerase; GPPS, geranyl diphosphate synthase; FPPS, farnesylpyrophosphate synthase; GGPPS, geranylgeranyl diphosphate synthase; MTPS, monoterpene synthase; STPS, sesquiterpene synthase; DTPS, diterpene synthase.

*S. glabra* produces oleoresin in particular tissues of stems known as secretory canals, where terpenes constitute a major component of oleoresin oil. Transcriptome analysis revealed sequences for a complete set of 60 genes in the terpene biosynthesis pathway in *S. glabra* (Figure 3). In the MVA pathway, transcriptome mining identified three, six and three putative genes for the initial three enzymes acetyl-CoA acetyltransferase (AACT), hydroxymethylglutaryl-CoA synthase (HMGS), and hydroxymethylglutaryl-CoA reductase (HMGR), respectively (Figure 3, File S7). Next, during the formation of IPP, the transcriptome analysis revealed two putative unigenes for mevalonate kinase (MVK), two for phosphomevalonate kinase (PMK), and two for mevalonate diphosphate decarboxylase (MVD). Among these unique genes, Cluster-32860.96052, encoding HMGS, exhibited higher expression in both H10 and H12 plants as compared to those in L4 and L6 plants, suggesting a possible role of this unigene in determining the terpene amount in *S. glabra*.

In the MEP pathway, generation of IPP or DMAPP involves seven enzymatic steps. Initially, a total of six and three putative genes were identified as 1-deoxy-D-xylulose-5-phosphate synthase (DXS) and 1-deoxy-D-xylulose-5-phosphate reductoisomerase (DXR), respectively (Figure 3, File S7). Next, the transcriptome exploration identified three unigenes for 2-C-methyl-D-erythritol 4-phosphate cytidylyltransferase (MCT), two for 4-diphosphocytidyl-2-C-methyl-D-erythritol kinase (CMK), two for 2-C-methyl-D-erythritol 2,4-cyclodiphosphate synthase (MDS), three for (E)-4-hydroxy-3-methylbut-2-enyl-diphosphate synthase (HDS), and three for 4-hydroxy-3-methylbut-2-enyl diphosphate reductase (HDR). Furthermore, the identified genes involved in the MVA pathway exhibited relatively higher expression compared to the MEP pathway (File S7), which is consistent with abundance of sesquiterpene compounds in the oleoresin of *S. glabra*.

The intermediate IPP can be isomerized into DMAPP by isopentenyl di-phosphate isomerase (IDI). The transcriptome analysis identified three representative unigenes for IDI (Figure 3, File S7). Geranyl diphosphate synthase (GPPS) catalyzes the condensation of IPP and DMPP to generate GPP, which is further utilized by farnesylpyrophosphate synthase (FPPS) for the synthesis of FPP that is subsequently catalyzed by geranylgeranyl diphosphate synthase (GGPPS) for the production of GGPP. The transcriptional mining identified two unigenes for GPPS, six for FPPS, and three for GGPPS. The relatively higher expression of FPPS unigenes than those of GPPS and GGPPS is in agreement with sesquiterpenes being the predominant component of terpene oil in *S. glabra*. Furthermore, Cluster-32860.104547, encoding FPPS in H10 and H12, exhibited an FPKM value eight- and five-fold higher than that of L4, respectively, indicating the potential influence of this gene on sesquiterpene yield.

For monoterpene biosynthesis, GPP was catalyzed by monoterpene synthases (MTPSs), such as geraniol synthase and linalool synthase, to produce different forms of monoterpene. However, there were no unigenes that contained a complete ORF for MTPSs discovered in these transcriptomes. For sesquiterpene biosynthesis, two putative genes were found to encode germacrene synthase (GS) and valencene synthase (VS), respectively (Figure 3, File S7). For diterpene biosynthesis, three and one genes were found to encode *ent*-copalyl diphosphate synthase (CPS) and *ent*-kaurene synthase (KS), respectively. In particular, Cluster-32860.192900, encoding STPS in H10, showed an FPKM value four-fold higher than that of L4, while Cluster-32860.116868, encoding STPS in H10 and H12, had an FPKM value six-fold higher than that of L4. These data indicated that the two identified STPSs in the terpene biosynthesis pathway may account for the variation in the terpene yield of *S. glabra*.

**Figure 4:**
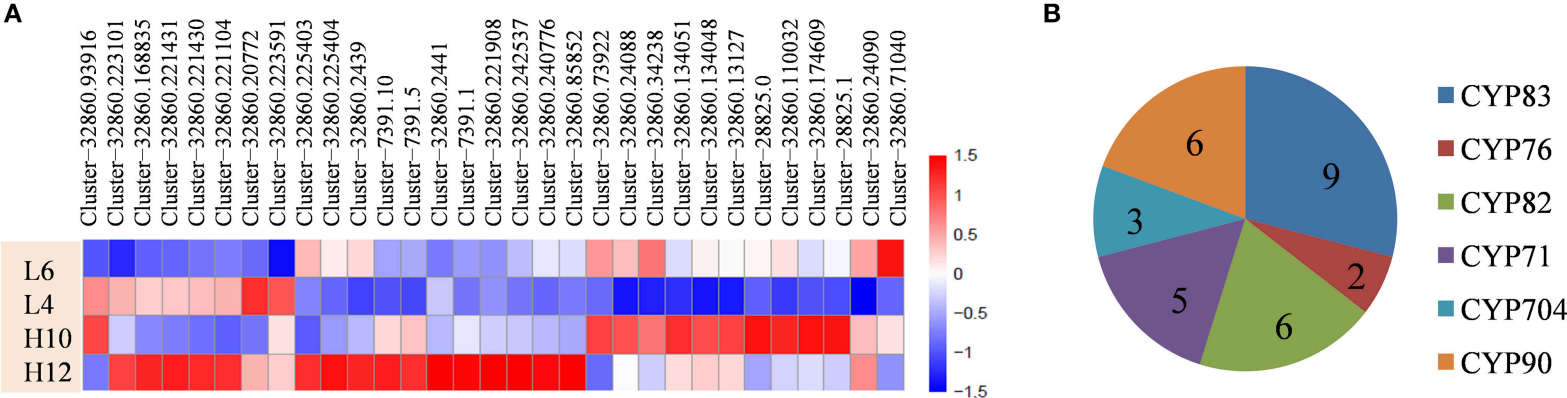
Differential expression profiles and distribution of CYPs genes. **(A)** Heat map showing the differential expression profile of CYPs. **(B)** Distribution of the CYP family. The scale represents log2 fold changes in H10 and H12 relative to L4 and L6. Unigenes with an adjusted *P*-value (*p*adj) < 0.05 were differentially expressed.

Cytochrome P450 family enzymes participate in the downstream modification of the terpene skeleton by regiospecific oxygenation and also contribute to another level of terpene diversity. Data analysis revealed that 31 candidate CYP genes were up-regulated in H10 or H12 compared to L4 and L6, with a maximum seven-fold FPKM change (Figure 4A, File S8). Depending on the phylogenetic analysis of the deduced protein sequence of these CYPs, six groups were identified, including nine unigenes in CYP83, two in CYP76, six in CYP82, five in CYP71, three in CYP704, and six in the CYP90 family (Figure 4B). Several CYPs, such as the CYP71 and CYP76 families, have been reported to catalyze the oxidation of sesquiterpenes in various plants (Diaz-Chavez et al., 2013; Takase et al., 2016). Therefore, the identified differentially expressed CYP genes could be of particular interest for further elucidation of terpene diversity in *S. glabra*.

### Transcription factors and hormone related genes

**Figure 5:**
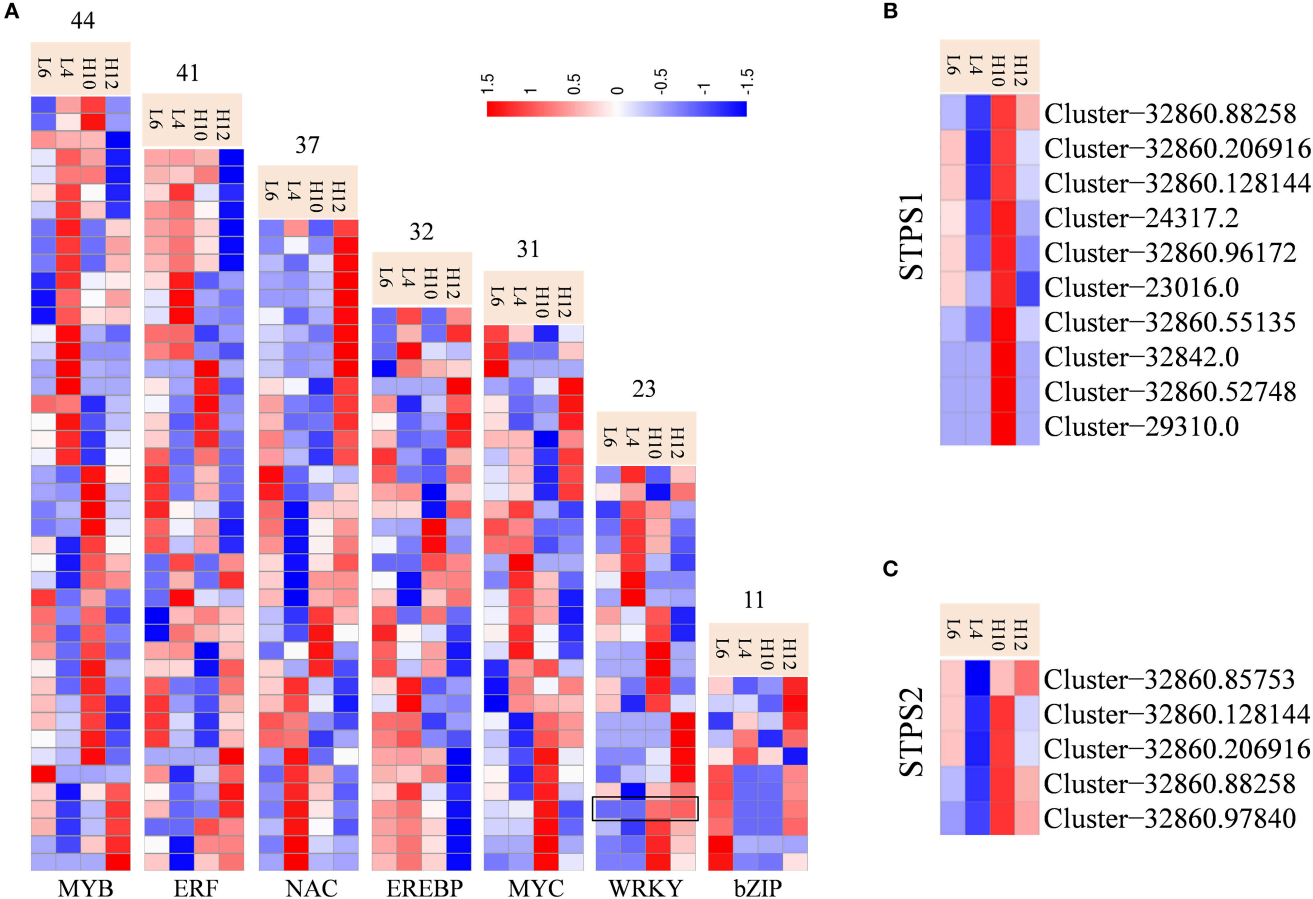
Differential expression profiles of transcriptional factors and co-expression analysis of STPSs and WRKY genes. **(A)** Heat maps representing transcriptional factors. The black box indicates the expression patterns of *SgWRKY*. **(B)** Co-expression of STPS1 and WRKY genes. **(C)** Co-expression of STPS2 and WRKY genes.

Transcription factors (TFs) have been characterized for regulating biosynthesis of terpene specialized metabolites in different plants, including AaWRKY, AaERF1, AaMYB1, AabZIP1, HbEREBP1, AsMYC2, and MsYABBY5 (Matías-Hernández et al., 2017; Xu et al., 2017). Transcriptome data identified abundant transcripts for putative TFs, among which MYB families were the most abundant (493), followed by MYC (283), NAC (272), WRKY (268), and ERF (256). There were 44 MYB, 41 ERF, 37 NAC, 32 EREBP, 31 MYC, 23 WRKY, and 11 bZIP unigenes identified as DEGs (Figure 5A, File S9). These could be potential regulators for metabolic engineering and improvement of the production of specialized metabolites in *S. glabra*. In particular, Cluster-32860.28516 annotated as WRKY (*SgWRKY*) was found to be up-regulated in both H10 and H12 compared to L4 and L6, indicating it as a possible positive regulator of terpene metabolism in *S. glabra*. Co-expression pattern analysis revealed that 10 and 5 WRKY genes were co-expressed with *STPS1* and *STPS2*, respectively (Figures 5B,C), which provides information for the possible genetic regulation network of terpenes in *S. glabra*.

Phytohormones, including JA, salicyclic acid (SA), and abscisic acid (ABA), have been identified as potential regulators of specialized metabolite biosynthesis (Zhou and Memelink, 2016). In the transcriptome data, a total of 422 unigenes were annotated as related to different hormones. Among them, nine for ethylene, eight for auxin, seven for ABA, six for brassinositol, four for cytokinin, three for SA, and two for JA pathway associated genes were detected as DEGs among samples (Figure S2, File S10). Cluster-32860.120273, encoding auxin response factor (*SgARF*), was down-regulated in both H10 and H12 compared with those of L4 and L6, indicating possible negative regulation of *SgARF* in terpene biosynthesis in *S. glabra*. These TFs and hormone related genes can provide foundations for investigating specialized metabolism as well as the engineering of terpene biosynthesis in *S. glabra*.

### Experimental qRT-PCR validation

To validate the expression of putative genes in RNA-seq data, 13 genes involved in the terpene biosynthesis pathway, including *AACT, HMGS, HMGR, MVK, PMK, MVD, MCT, CMK, MDS, HDS, HDR, IDI*, and *GGPS*, were selected for qRT-PCR analysis. The comparative analysis of these genes obtained by qRT-PCR revealed similar expression patterns as those obtained by transcriptome analysis (Figure S3A). Statistical analysis showed a strong correlation between the qRT-PCR results and the dataset obtained by RNA-seq; the correlation coefficient was 0.942 (Figure S3B), suggesting that the data obtained by transcriptome sequencing are reliable for exploring the target genes and regulatory genes involved in terpene biosynthesis.

### Expression patterns of key genes

**Figure 6:**
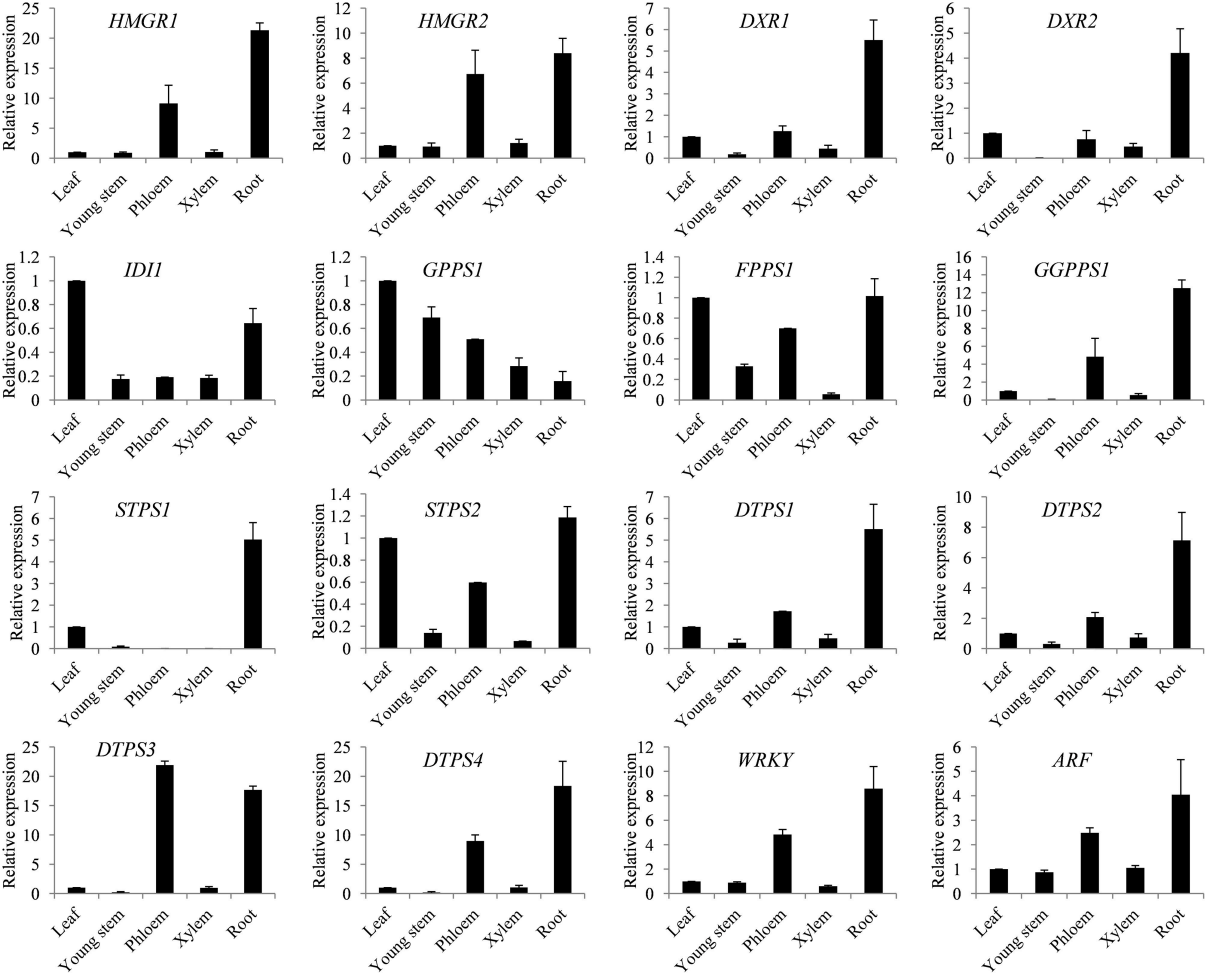
Spatial expression patterns of key genes in the terpene synthesis pathway. Expression of genes from the leaf, young stem, phloem, xylem and root was examined by qRT-PCR. Abbreviations for genes are the same as in Figure 4. *SgActin* was used as an internal control. Data are presented as the mean ± SE of triplicate samples.

In order to gain insight into the spatial expression patterns of genes involved in terpene biosynthesis in *S. glabra*, the expression levels of 16 key identified genes were analyzed by qRT-PCR in different tissues, including the leaf, young stem, phloem and xylem from the trunk, and root. The putative rate-limiting genes *HMGR1* and *HMGR2* from MVA, and *DXR1* and *DXR2* from the MEP pathways showed higher expression in the phloem and root with relatively low expression in the leaf, young stem, and xylem (Figure 6). The diterpene synthesis genes including *GGPPS1* and diterpene synthases *DTPS1, DTPS2, DTPS3*, and *DTPS4* all exhibited preferential expression in the phloem and root. The putative transcription factors *WRKY* and *ARF* were also mainly expressed in the phloem and root. The two sesquiterpene synthase genes *STPS1* and *STPS2* were differentially regulated in various tissues, among which *STPS1* showed the highest expression in the root, and *STPS2* was mainly expressed in the leaf, phloem, and root. These expression patterns of genes were consistent with the terpene profile that terpene compounds mainly accumulate in the trunk of *S. glabra* plants.

### Phylogenetic analysis of SgTPSs

**Figure 7:**
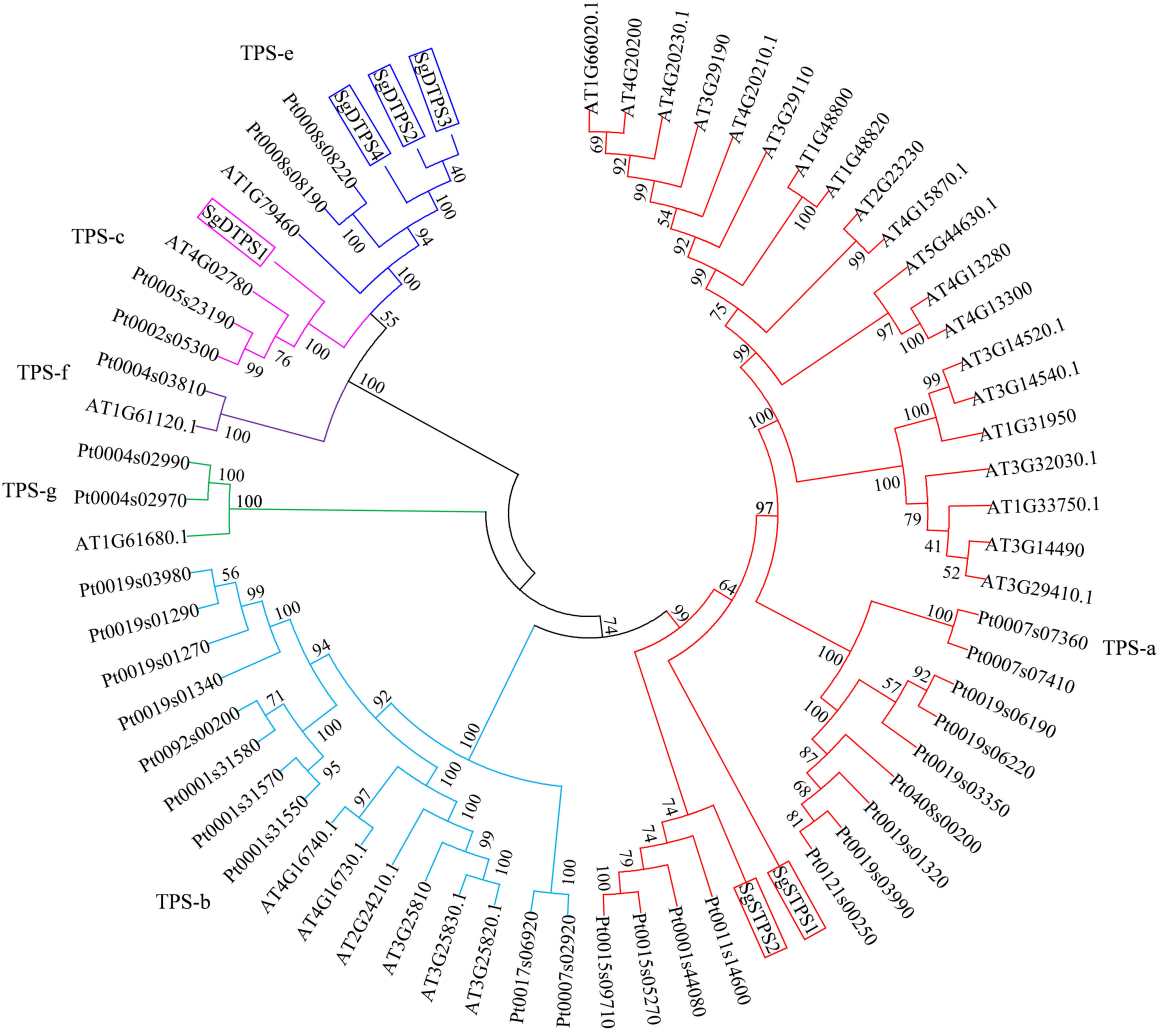
Phylogenetic analysis of terpene synthases of *S. glabra* relative to those of *Arabidopsis thaliana* and *Populus trichocarpa*.

The TPSs family can be classified into six clades including TPS a-g (Chen et al., 2011). To clarify the function of TPS in *S. glabra*, we selected putative TPS genes that contained a complete ORF sequence and encoded proteins larger than 500 amino acids. Two STPS transcripts, Cluster-32860.192900 (SgSTPS1, 549 aa), and Cluster-32860.116868 (SgSTPS2, 557 aa), and four DTPS transcripts, Cluster-32860.70817 (SgDTPS1, 597 aa), Cluster-32860.241723 (SgDTPS2, 789 aa), Cluster-32860.433 (SgDTPS3, 762 aa), and Cluster-24327.0 (SgDTPS4, 686 aa), were then identified. Blast search of SgSTPS1 and SgSTPS2 showed closest homology to CoTPS1 (AGW18154) (93%) and CoTPS4 (AGW18157) (89%) from *Copaifera officinalis*, respectively; both of them were functionally characterized as sesquiterpene synthase (Joyce 2013). SgDTPS1 was closely related to CoTPS5 (AGW18158) (88%) and TcCPS (EOX94746) (57%) from *Theobroma cacao*, while SgDTPS2, SgDTPS3, and SgDTPS4 were most closely related to GsKS (KHN21375) (about 71%) from *G. soja*. Multiple sequence alignment revealed that SgSTPS1 and SgSTPS2 contained the RRX_8_W, EDXXD, DDXXD, and NSE/DTE motifs (File S11). SgDTPS1 was aligned with other CPSs and was found to include the QXXDGGWG and DXDDTAM motifs, suggesting it as a class II terpene synthase. SgDTPS2, SgDTPS3, and SgDTPS4 were aligned with other KSs and were revealed to contain the QXXDGGWG, DDXXD, and NSE/DTE motifs. The six genes were further used to build phylogenetic relationships with other characterized TPSs. It was found that SgSTPS1 and SgSTPS2 were all clustered into the TPS-a subfamily, suggesting they were sesquiterpene synthases (Figure 7). SgDTPS1 was grouped together with CPS in TPS-c subfamily, suggesting this as a class II diterpene cyclase. SgDTPS2, SgDTPS3, and SgDTPS4 were grouped with KS in the TPS-e subfamily, indicating them to be diterpene synthase. These genes can serve as key candidates for investigating the biological function of terpene synthases in *S. glabra*.

### Subcellular localization of SgSTPSs

**Figure 8:**
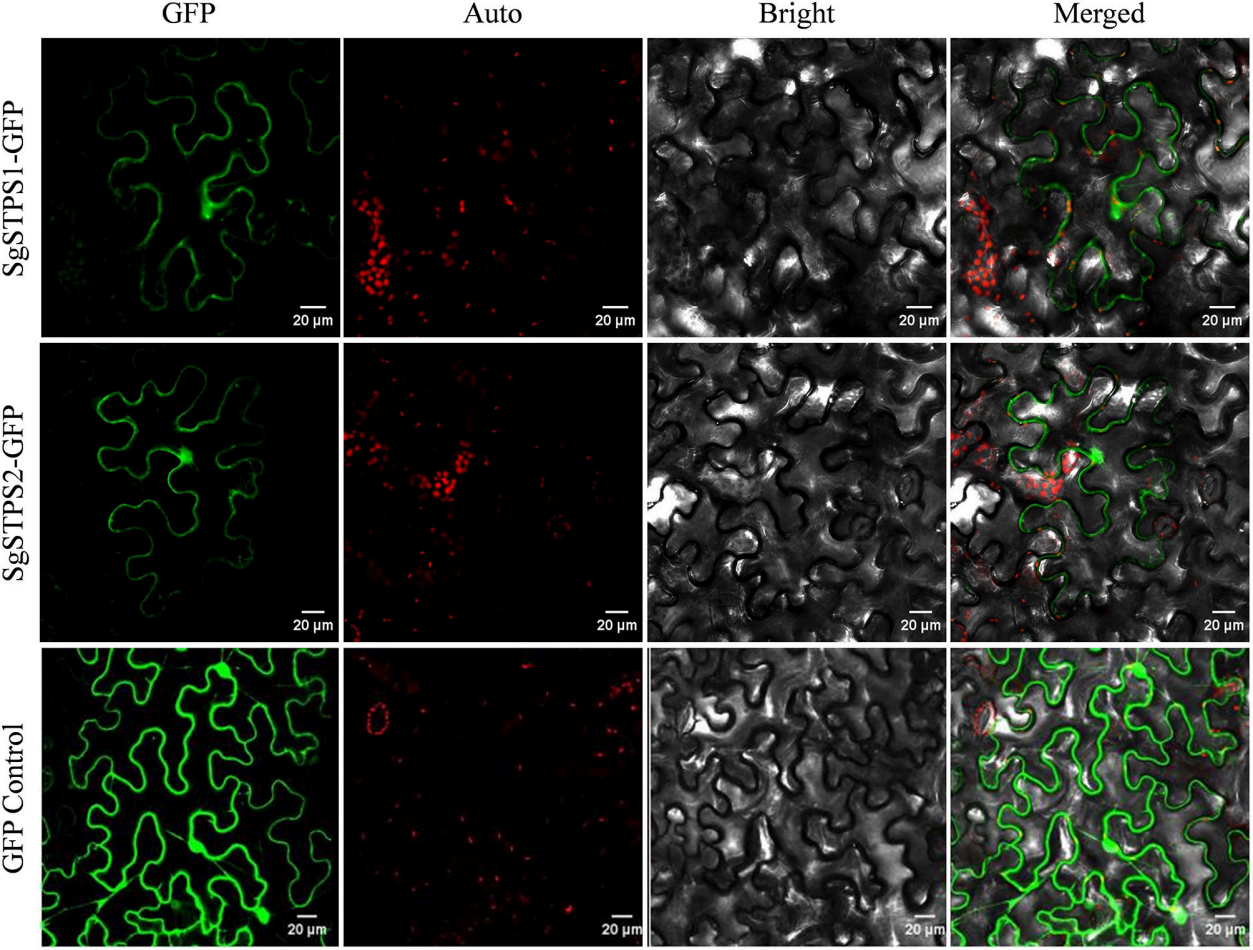
Subcellular localization of *S. glabra* sesquiterpene synthases. Confocal laser scanning microscopy of SgSTPS1 and SgSTPS2 using GFP-fusion proteins in *Nicotiana benthamiana*. GFP, GFP fluorescene; Auto, chlorophyll autofluorescene; Merged, combined GFP and chlorophyll autofluorescene. Scale bars = 20 μm.

Since the majority (85%) of compounds in *S. glabra* oleoresin belong to sesquiterpenes, the two identified sequiterpene synthases, SgSTPS1 and SgSTPS2, were subject to further functional analysis. *In silico* signal peptide prediction by SignalIP showed that the two SgSTPSs were cytosolic with no signal peptide detected, which correlated well with the prediction of SgSTPS1 and SgSTPS2 as sesqui-TPSs. To understand the biological function, the full-length sequences of *SgSTPS1* and *SgSTPS2* genes were obtained by cloning using the cDNA library of *S. glabra* stem as a template and were confirmed by sequencing to have the exact sequence as those identified by transcriptome sequencing. To test the subcellular localization, the two genes *SgSTPS1* and *SgSTPS2* were fused in frame with the GFP reporter gene and were transformed into *N. benthamiana*. Confocal analysis revealed the SgSTPS1-GFP and SgSTPS2-GFP proteins were localized in the cytoplasm (Figure 8), suggesting that SgSTPS1 and SgSTPS2 are responsible for sesquiterpene production in the cytosol.

### Biochemical function of SgSTPSs

**Figure 9:**
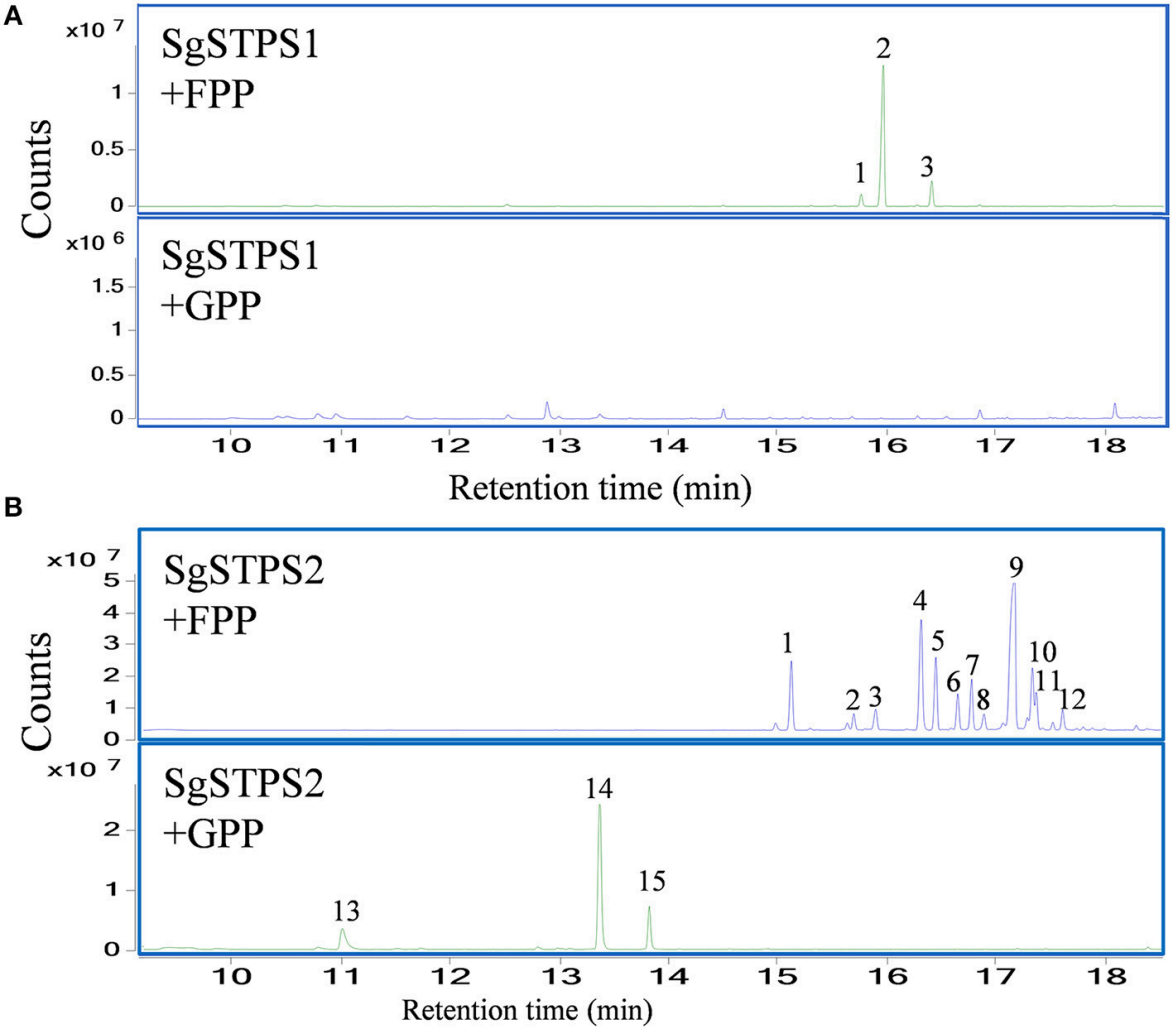
*In vitro* characterization of recombinant SgSTPS enzymes using FPP or GPP as a substrate. **(A)** GC-MS chromatograms for SgSTPS1. Peak 1, isocaryophillene; Peak 2, β-caryophyllene; Peak 3, humulene. **(B)** GC-MS chromatograms for SgSTPS2. Peak 1, elemene isomer; Peak 2, α-copaene; Peak 3, β-elemene; Peak 4, ylangene; Peak 5, β-copaene; Peak 6, isogermacrene D; Peak 7, γ-cadinene; Peak 8, γ-muurolene; Peak 9, germacrene D; Peak 10, bicyclogermacrene; Peak 11, γ-amorphene; Peak 12, cadina-1(10),4-diene; Peak 13, linalool; Peak 14, geranyl methyl ether; Peak 15, geraniol.

To characterize the function of the sequiterpene synthases SgSTPS1 and SgSTPS2, the recombinant proteins SgSTPS1 and SgSTPS2 were expressed in *E. coli*, and purified proteins were used for activity assay. GC-MS analysis of the SgSTPS1 enzymatic products revealed the formation of predominantly β-caryophyllene (Figure 9A, Figure S4A), along with a minor amount of isocaryophillene and humulene when utilizing FPP as a substrate. While using GPP as a substrate, there were trace amounts of linalool and geraniol produced at the retention times of 10.42 and 13.37 min, respectively (Figure S5). This indicated that SgSTPS1 is a sesquiterpene synthase with caryophyllene synthase activity that could be responsible for β-caryophyllene production in *S. glabra*.

However, the recombinant SgSTPS2 protein was found to be a versatile enzyme that used FPP as a substrate to catalyze the synthesis of 12 sesquiterpene compounds, including elemene isomer, α-copaene, β-elemene, ylangene, β-copaene, isogermacrene D, γ-cadinene, γ-muurolene, germacrene D, bicyclogermacrene, γ-amorphene, and cadina-1(10),4-diene (Figure 9B, Figure S4B), all of which were also present in *S. glabra* oleoresin except elemene (Figure 1). Among the sesquiterpene products, elemene, ylangene, β-copaene, and germacrene D were the major products. When using GPP as a substrate, SgSTPS2 synthesized three acyclic monoterpenes, linalool, geranyl methyl ether, and geraniol, demonstrating MTPSs activity of SgSTPS2.

## Discussion

### Transcriptome analysis and terpene variation in *S. glabra*

In an attempt to investigate the molecular basis of terpene biosynthesis in *S. glabra*, an RNA-seq approach was employed to sequence the stem transcriptome from high and low oil-yielding trees. After transcriptome assembly, the total number, N50 length and mean length of *de novo* assembled transcipts and unigenes of *S. glabra* were much higher than those recently reported in other non-model plants including *Carya illinoinensis* (Mo et al., 2018), *Kandelia obovata* (Hong et al., 2018), *Kalopanax septemlobus* (Han et al., 2018), *Salvia officinalis* (Ali et al., 2017), and *Melaleuca alternifolia* (Bustos-Segura et al., 2017), suggesting high accuracy and reliability of the sequencing data. Numerous annotated unigenes (61.9%) were found to be distributed in the Fabaceae family, which was reasonable since *S. glabra* belongs to the Caesalpinioideae subfamily that is included in the Fabaceae family. Both GO and KEGG enrichment analysis of DEGs in H10 and H12 compared to L4 and L6 revealed that the terpene biosynthesis process and the plant hormone signal transduction pathway may play roles in determining the terpene variation in *S. glabra*. However, even plants belonging to the same type (low- or high-producing type) exhibited enormous differences in gene expression profiles (Figure 2A). These DEGs between H10 and H12 or between L4 and L6 may be accountable for the difference in the ratio of different terpene compounds (Figure 1A).

### Evolutionary origin of SgTPSs

Based on the reaction mechanism and products formed, plant TPSs can be classified as class I, class II, or class I/II enzymes. Class I TPSs contain the DDXXD and NSE/DTE motifs that coordinate with Mg^2+^ on their C-terminus. Class II TPSs include the DXDD motif for protonation-initiated cyclization of the substrate. The bifunctional class I/II diTPS harbors both functional active sites (Chen et al., 2011). In *S. glabra*, the sesquiterpene synthases, SgSTPS1 and SgSTPS2, and the diterpene synthases, SgDTPS2, SgDTPS3, and SgDTPS4, contain the DDXXD and NSE/DTE motifs, suggesting that they are class I TPSs that require Mg^2+^ as a cofactor. The sequence annotation, homology and phylogenetic analysis indicated that both SgSTPS1 and SgSTPS2 are sesquiterpene synthases. However, both SgSTPS1 and SgSTPS2 contain an additional EDXXD motif at the N-terminus, which in its active form contributes to class II diterpene synthase activity (Cao et al., 2010). Bifunctional class I/II diterpene synthases are only known in non-vascular plants and gymnosperms. In angiosperms, all of the diterpene synthases that have been characterized to date are monofunctional, with loss of activity in one domain or the other. All of the sesquiterpene synthases are monofunctional, having retained only one active site. TPSs containing the EDXXD domain are members of the TPS-d, -c, -e, -f clades (Martin et al., 2004). Furthermore, an additional RRX_8_W motif was also found at the N-terminus of the two SgSTPS genes, but it was not present in the four SgDTPS genes. The RRX_8_W motif is essential for cleavage of the transit peptide in mono- and di-terpene synthases (Chen et al., 2011). Subcellular localization confirmed that the two SgSTPSs were localized in the cytosol (Figure 8). *S. glabra* terpene synthase gene motifs may suggest the evolutionary origin of terpene synthase through domain loss or subfunctionalization from a common ancestor, which has been reported in *Magnolia* sesquiterpene synthase (Lee and Chappell, 2008) and *Copaifera* sesquiterpene synthases (Joyce, 2013). This hypothesis was further supported by the enzymatic activity of the two SgSTPSs. Although SgSTPS1 mainly catalyzed the formation of sesquiterpene caryophyllene when using FPP as substrate, there were trace amounts of linalool and geraniol produced at the retention times of 10.42 and 13.37 min, respectively, when using GPP as the substrate (Figure S5). SgSTPS2 was confirmed to have bi-substrate capability that could catalyze both GPP and FPP to produce monoterpenes and sesquiterpenes, respectively (Figure 9B). These results indicated that SgSTPS1 and SgSTPS2 still retain partial MTPSs activity. Multi-substrate capability was found to be usual in MTPSs and STPSs (Pazouki and Niinemets, 2016). However, there was no monoterpene discovered in *S. glabra* oleoresin and no chloroplastic signal peptide could be detected in SgSTPS1 and SgSTPS2. As monoterpenes are usually produced in chloroplasts through the MEP pathway, these results indicate that *S. glabra* sesquiterpene synthases may evolve through the loss of the chloroplast signal peptide. Further functional and structural characterizations are needed to decipher the evolutionary shift from mono- and di-terpene-rich oleoresin in gymnosperms to sesquiterpene-abundant oleoresin in angiosperms.

### Contribution of SgSTPSs to *S. glabra* oleoresin composition

It has been reported that the transcript abundance of TPS is positively correlated with the accumulation of terpene (Nieuwenhuizen et al., 2009; Bustos-Segura et al., 2017; Yu et al., 2017). In this study, the two identified SgSTPSs revealed higher expression in H10 compared to L4, suggesting the potential roles of the two genes in determining the terpene amount in *S. glabra*. In *S. glabra* oleoresin, the major components of sesquiterpenes are α-copaene (32.26%) and β-caryophyllene (16.33%) (Figure 1, Yang et al., 2016). Here, by a combination of transcriptome and experimental analyses, we identified that SgSTPS1 was mainly responsible for β-caryophyllene sesquiterpene production, while SgSTPS2 was accountable for α-copaene production. Nevertheless, SgSTPS2 is a versatile enzyme that can also produce other sesquiterpenes including ylangene, germacrene, elemene, cadinene, muurolene, and amorphene, all of which were present in *S. glabra* oleoresin except elemene, which may be a byproduct due to rearrangements of different sesquiterpenes (Agger et al., 2008). Therefore, the two SgSTPS enzymatic products matched well with the chemical composition of oleoresin in the *S. glabra* stem. Furthermore, these results indicated that different sesquiterpene synthases have diversified functionally to produce specific kinds of products. Among the characterized plant β-caryophyllene synthases, SgSTPS1 exhibited the highest amino acid identity (46%) to GhTPS from *Gossypium hirsutum* (AFQ23183) (Huang et al., 2013), 40% identity to AtTPS from *A. thaliana* (AAO85539) (Huang et al., 2012), and the lowest identity (35%) to ZmTPS from *Zea mays* (ABY79207) (Köllner et al., 2008), three of which catalyzed the formation of β-caryophyllene as the major product from FPP. In particular, the product amorphadiene that SgSTPS2 synthesizes from FPP is a precursor of the antimalarial drug artemisinin, which can be used for the production of artemisinic acid. The identified SgSTPS2 shared 42% similarity with amorpha-4,11-diene synthase from *A. annua* (ABM88787). Furthermore, β-caryophyllene and α-copaene in plant species were found to serve in defense against pathogens or herbivores, and the identified sesquiterpene synthases were involved in the defense response (Huang et al., 2012). Therefore, we hypothesize that SgSTPSs may be responsible for the defense response in *S. glabra*. Further research is needed to investigate the ecological functions of sesquiterpenes and corresponding SgSTPSs.

### Potential regulatory network of terpene synthesis

TFs play a predominant role in regulating the expression of genes in various metabolic pathways. Identification of those TFs could be important for understanding the regulatory mechanism of terpene biosynthesis in *S. glabra*. Most of the identified TFs for regulating terpene metabolites belong to the WRKY family, including *TcWRKY1* from *Taxus chinensis* (Li S. et al., 2013), *AaWRKY1* from *A. annua* (Ma et al., 2009), *GaWRKY1* from *Gossypium arboreum* (Xu et al., 2004), and *HbWRKY1* from *Hevea brasiliensis* (Wang et al., 2013), which activate the expression of genes encoding key enzymes involved in the metabolite pathway. Here, we suggest that *SgWRKY* may be a positive regulator in *S. glabra* terpene biosynthesis (Figure 5A), which is consistent with the role of WRKY in other plants based on previous research. However, the regulatory role and downstream target of *SgWRKY* in terpene biosynthesis need to be further explored and confirmed by functional analysis. More transcriptome and experimental data are needed to elucidate the metabolic regulatory network. How transcription factors are connected to the signaling pathway and how their action is integrated with other regulatory circuits await further investigation in *S. glabra*.

## Author contributions

NY designed the study and wrote the manuscript. NY, J-CY, and R-SL performed the experiments. G-TY and W-TZ helped in data analysis and manuscript preparation.

### Conflict of interest statement

The authors declare that the research was conducted in the absence of any commercial or financial relationships that could be construed as a potential conflict of interest.

## Supplementary material

Figure S1 Functional annotation of unigenes from *S. glabra* transcriptome against databases. **(A)** Venn diagram showing the number of unigenes annotated in five databases. **(B)** Species classification against the nr database for all annotated unigenes. **(C)** GO classification of all unigenes. **(D)** KOG classification of all unigenes. **(E)** KEGG classification of all unigenes.

Figure S2 Differential expression profiles of genes related to hormones involved in terpene biosynthesis. Black box indicates expression patterns of *SgARF*.

Figure S3 qRT-PCR validation of differentially expressed genes in RNA-seq dataset. **(A)** Expression levels of genes by qRT-PCR and in RNA-seq. Error bars indicate standard error. **(B)** Correlation analysis of expression results from qRT-PCR and RNA-seq.

Figure S4 Mass spectra for the peaks in GC-MS for the products formed by SgSTPS1 **(A)** and SgSTPS2 **(B)**. The peaks marked with numbers were identified by comparing with mass spectra library. The mass spectra for the peaks are shown with the references.

Figure S5 GC-MS chromatogram for the products formed by SgSTPS1 enzyme using GPP as substrate. The peaks marked with numbers were identified by comparing with mass spectra library. The mass spectra for the peaks are shown on the right and lower sides with the references.

Table S1 Summary of RNA-Seq data from all samples.

Table S2 Summary of assembled transcripts and unigenes.

Table S3 Statistics of unigene annotation rate.

File S1 Details of species classification of all annotated unigenes.

File S2 Details of GO classifications of all annotated unigenes.

File S3 Details of KOG classifications of all annotated unigenes.

File S4 Details of KEGG classifications of all annotated unigenes.

File S5 Details of identified DEGs involved in terpene pathway.

File S6 Details of all down-regulated and up-regulated DEGs among samples.

File S7 Details of DEGs involved in terpene biosynthesis pathway.

File S8 Details of differentially expressed CYPs genes.

File S9 Details of differentially expressed transcription factors involved in regulating terpene biosyntheis in *S. glabra*.

File S10 Details of differentially expressed hormone related genes involved in regulating terpene biosynthesis in *S. glabra*.

File S11 Multiple sequence alignment of *S. glabra* TPS genes with homologous genes from other plants.

File S12 Primers used in qRT-PCR validation of expression levels of genes.
