# Supplementary figures and images for: Transcriptome Analysis of Oleoresin-Producing Tree Sindora Glabra and Characterization of Sesquiterpene Synthases
Source: Front Plant Sci. 2018 Nov 20;9:1619. doi: 10.3389/fpls.2018.01619 (PMC6256070; doi:10.3389/fpls.2018.01619)

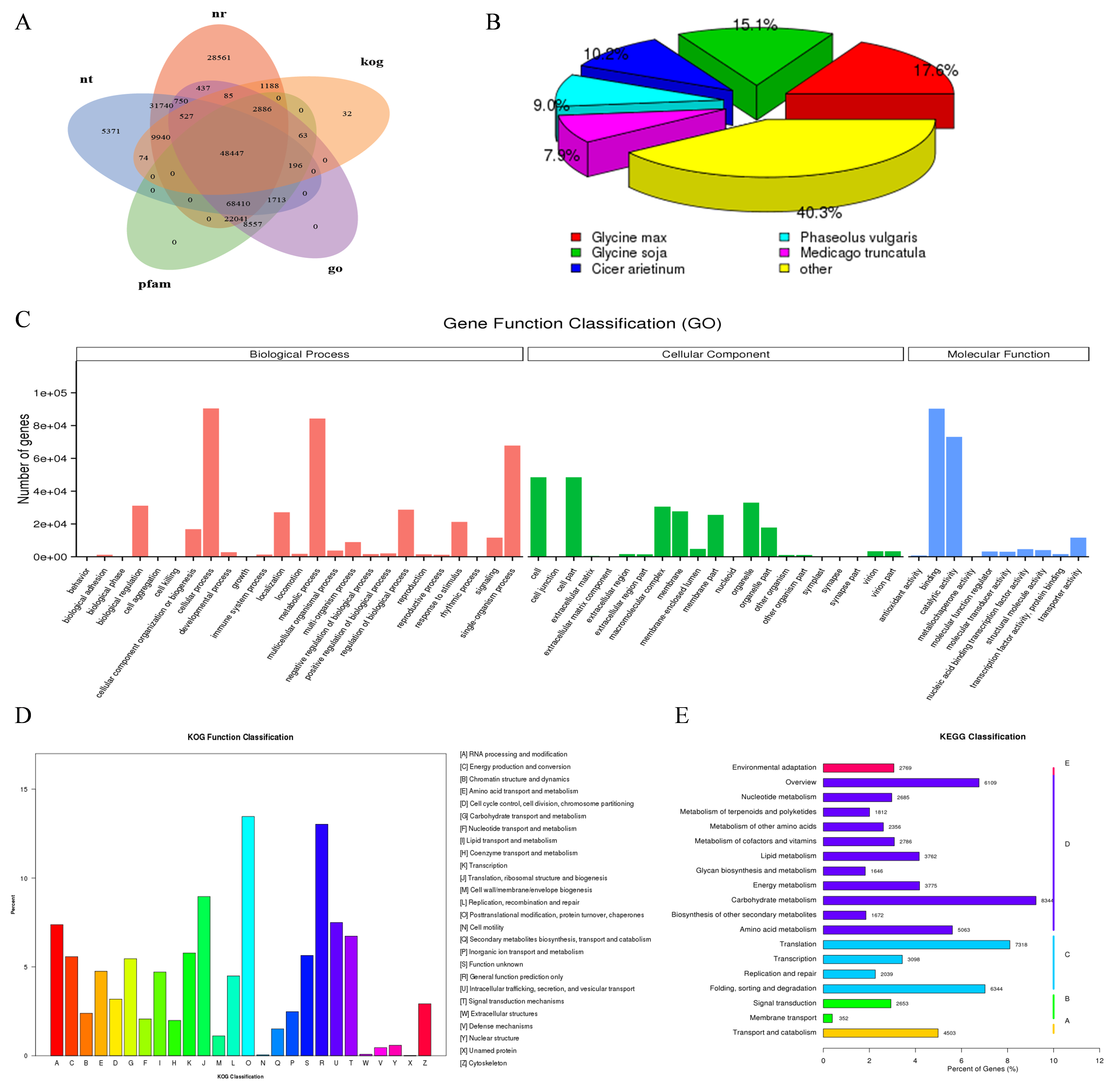

Supplement: Figure S1 — Functional annotation of unigenes from S. glabra transcriptome against databases. (A) Venn diagram showing the number of unigenes annotated in five databases. (B) Species classification against the nr database for all annotated unigenes. (C) GO classification of all unigenes. (D) KOG classification of all unigenes. (E) KEGG classification of all unigenes. [file Image_1.TIF]

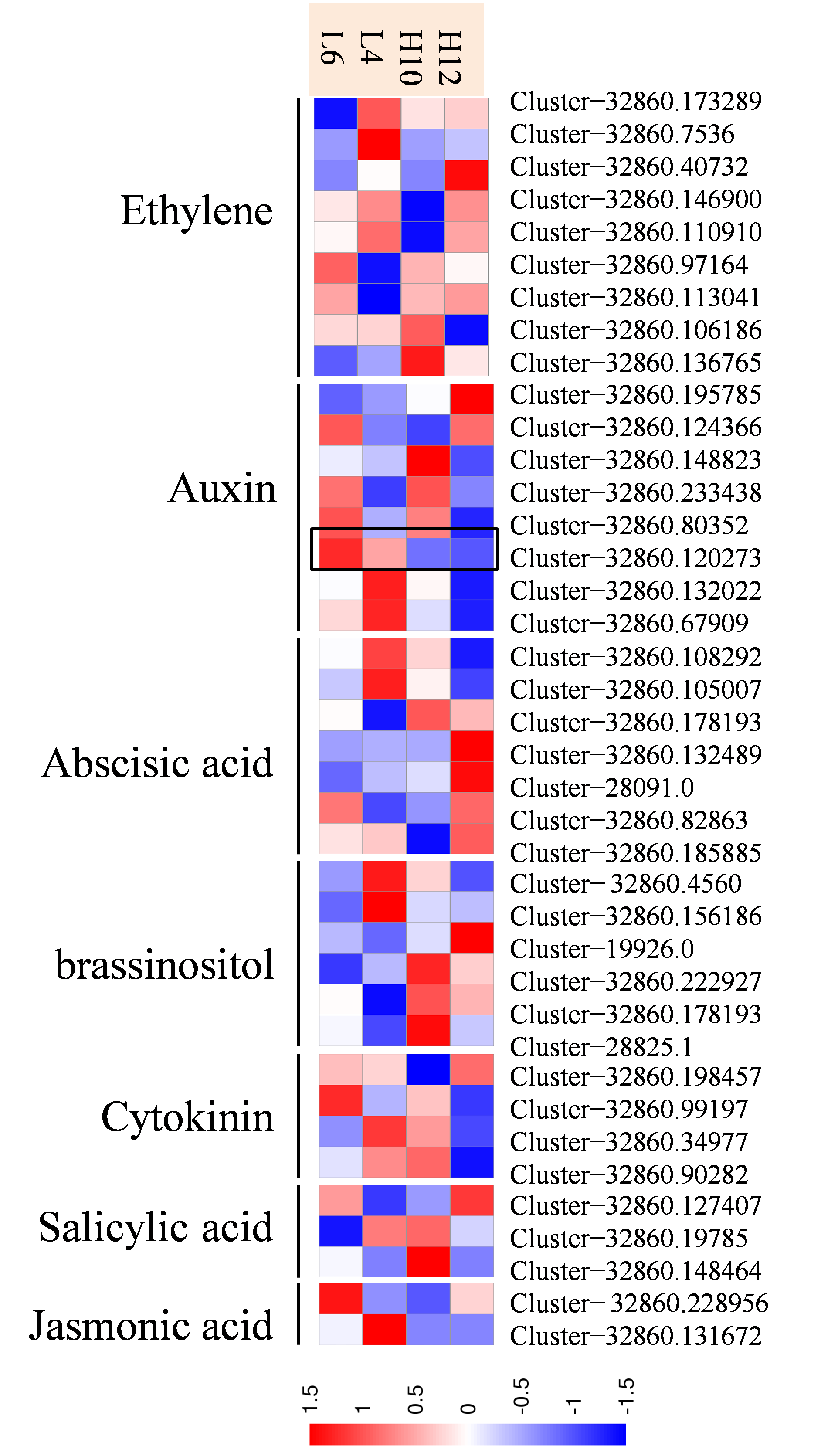

Supplement: Figure S2 — Differential expression profiles of genes related to hormones involved in terpene biosynthesis. Black box indicates expression patterns of SgARF. [file Image_2.TIF]

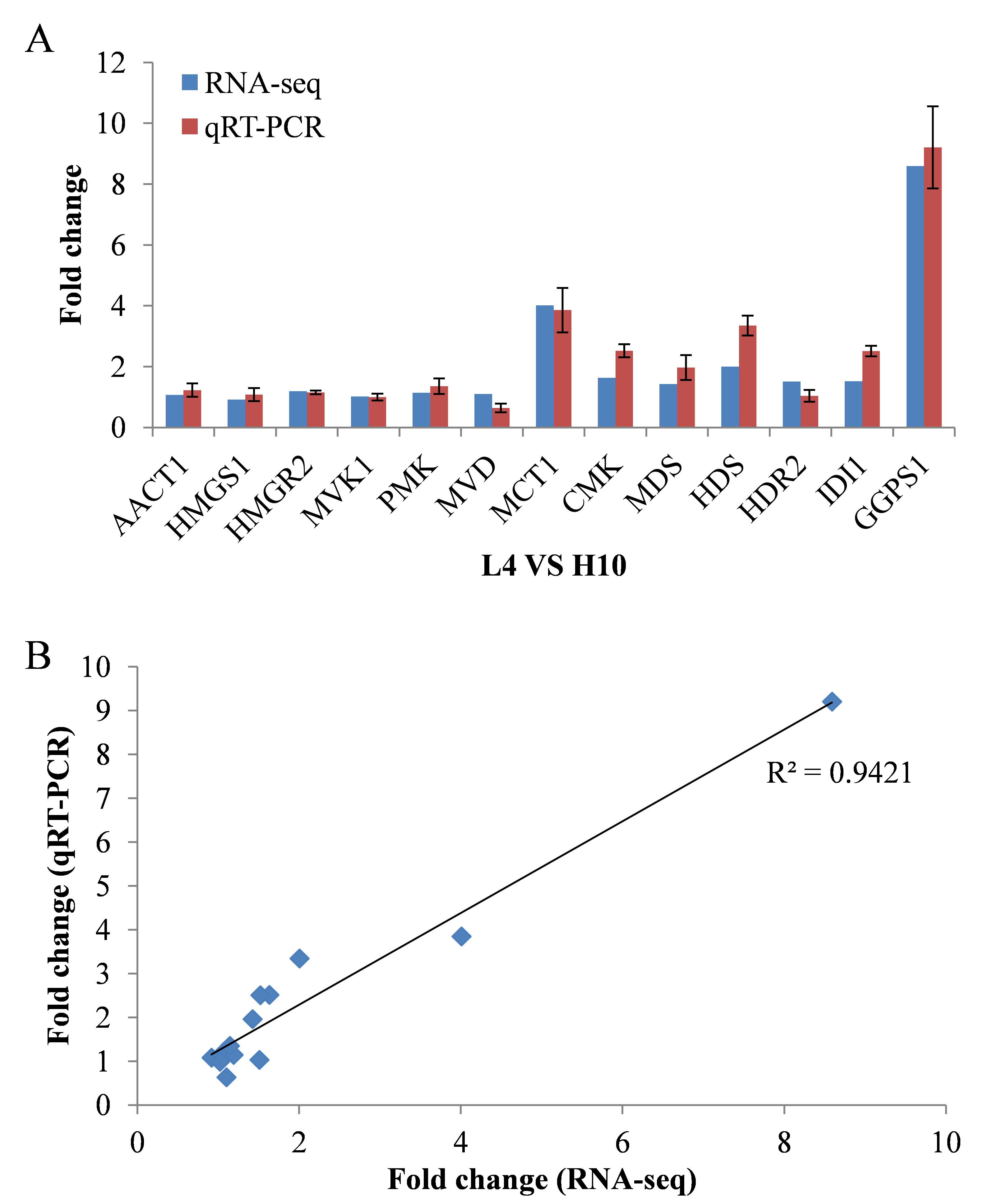

Supplement: Figure S3 — qRT-PCR validation of differentially expressed genes in RNA-seq dataset. (A) Expression levels of genes by qRT-PCR and in RNA-seq. Error bars indicate standard error. (B) Correlation analysis of expression results from qRT-PCR and RNA-seq. [file Image_3.TIF]

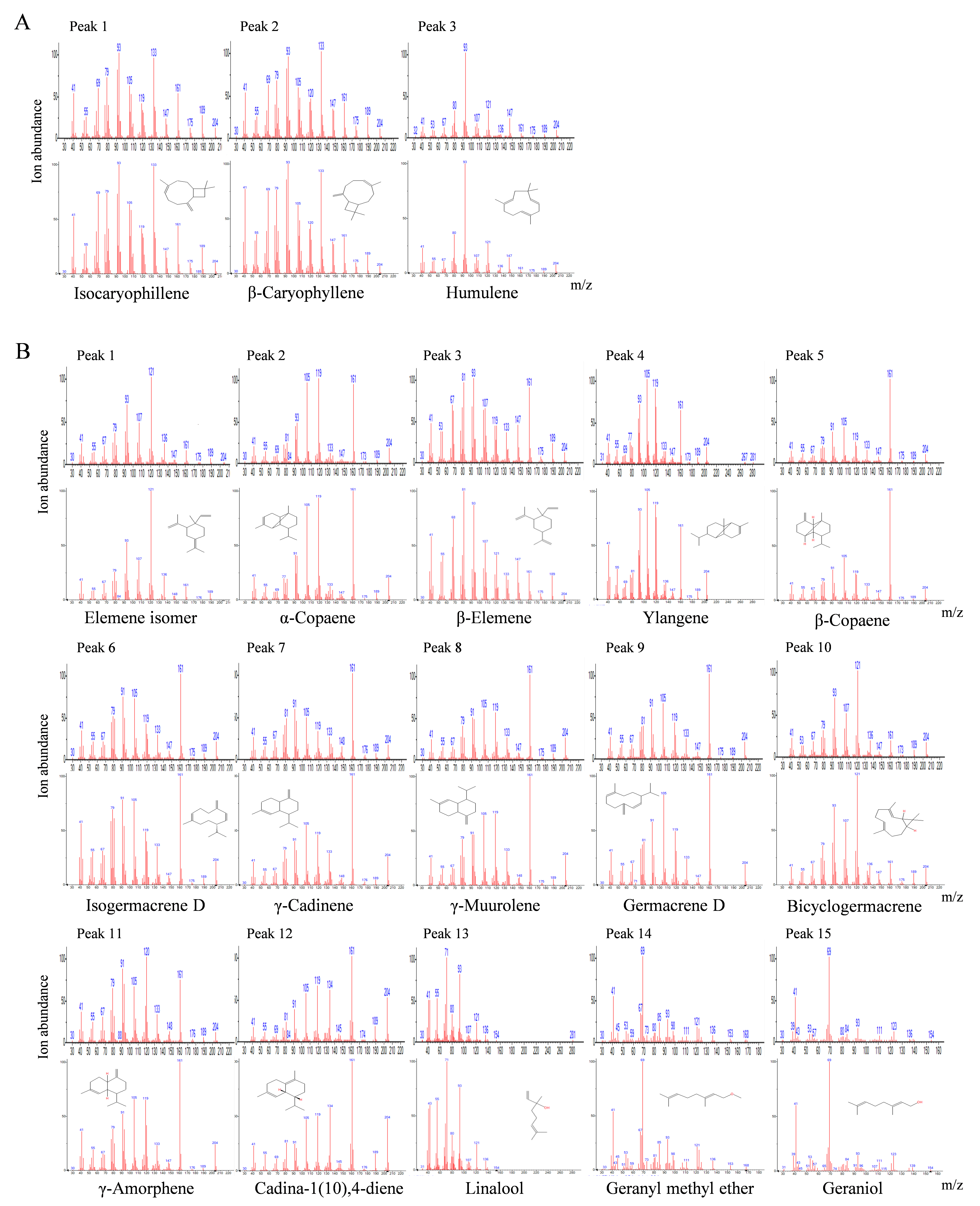

Supplement: Figure S4 — Mass spectra for the peaks in GC-MS for the products formed by SgSTPS1 (A) and SgSTPS2 (B). The peaks marked with numbers were identified by comparing with mass spectra library. The mass spectra for the peaks are shown with the references. [file Image_4.TIF]

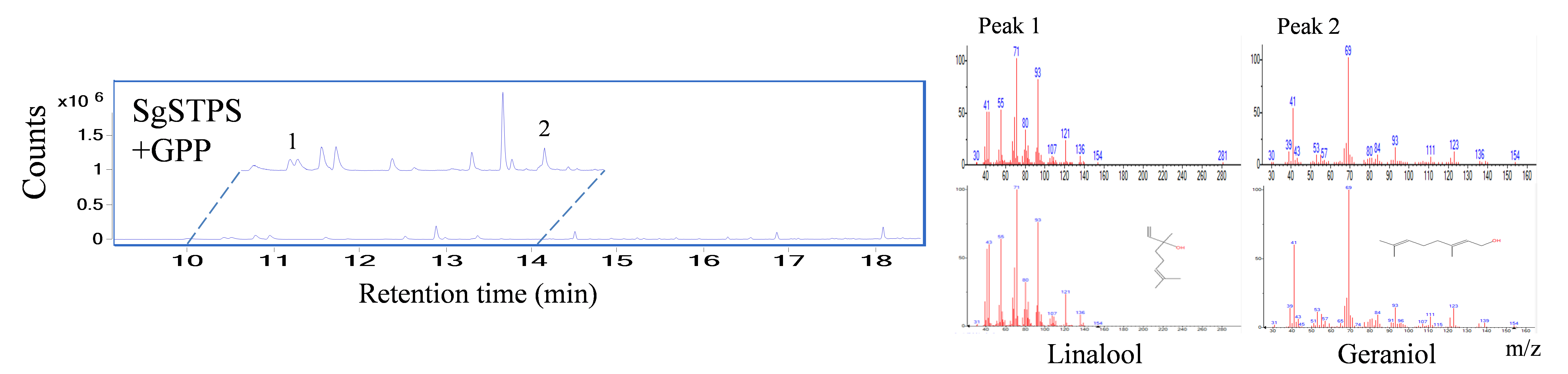

Supplement: Figure S5 — GC-MS chromatogram for the products formed by SgSTPS1 enzyme using GPP as substrate. The peaks marked with numbers were identified by comparing with mass spectra library. The mass spectra for the peaks are shown on the right and lower sides with the references. [file Image_5.TIF]
