# Supplementary material for: Transcriptome Analysis of Oleoresin-Producing Tree Sindora Glabra and Characterization of Sesquiterpene Synthases
Source: Front Plant Sci. 2018 Nov 20;9:1619. doi: 10.3389/fpls.2018.01619 (PMC6256070; doi:10.3389/fpls.2018.01619)
Supplement: Table S1 — Summary of RNA-Seq data from all samples. [file Table_12.DOCX]

**Supplemental tables**

**Table S1. Summary of RNA-Seq data from all samples.**

| **Sample** | **Raw Reads** | **Clean reads** | **Clean bases** | **Error(%)** | **Q20(%)** | **Q30(%)** | **GC(%)** |
| --- | --- | --- | --- | --- | --- | --- | --- |
| L4_1 | 59,165,190 | 57,686,990 | 8.65G | 0.01 | 97.58 | 93.83 | 43.68 |
| L4_2 | 49,328,008 | 47,924,644 | 7.19G | 0.01 | 97.34 | 93.38 | 43.48 |
| L4_3 | 54,762,914 | 53,248,772 | 7.99G | 0.01 | 97.45 | 93.62 | 43.65 |
| L6_1 | 44,009,170 | 42,759,508 | 6.41G | 0.01 | 97.47 | 93.64 | 43.66 |
| L6_2 | 68,783,074 | 66,910,394 | 10.04G | 0.01 | 97.45 | 93.61 | 43.67 |
| L6_3 | 43,595,146 | 42,334,318 | 6.35G | 0.01 | 97.38 | 93.47 | 43.35 |
| H10_1 | 54,580,160 | 53,020,860 | 7.95G | 0.01 | 97.55 | 93.80 | 43.63 |
| H10_2 | 52,961,840 | 51,524,856 | 7.73G | 0.01 | 97.40 | 93.50 | 43.69 |
| H10_3 | 56,668,940 | 55,071,586 | 8.26G | 0.01 | 97.51 | 93.69 | 43.67 |
| H12_1 | 63,240,774 | 61,315,346 | 9.2G | 0.01 | 97.70 | 94.07 | 43.72 |
| H12_2 | 58,029,220 | 56,442,798 | 8.47G | 0.01 | 97.62 | 93.91 | 43.65 |
| H12_3 | 61,045,596 | 59,400,446 | 8.91G | 0.01 | 97.48 | 93.64 | 43.73 |

**Table S2. Summary of assembled transcripts and unigenes.**

|  | **Transcripts** | **Unigenes** |
| --- | --- | --- |
| 200-500 bp | 170,282 (41.62%) | 49,851 (17.55%) |
| 500-1k bp | 69,435 (16.97%) | 65,123 (22.93%) |
| 1k-2k bp | 77,630 (18.98%) | 77,294 (27.22%) |
| >2k bp | 91,759 (22.43%) | 91,730 (32.30%) |
| Total unmber | 409,106 | 283,998 |
| Min Length | 201 | 201 |
| Mean Length | 1,270 | 1,701 |
| Median Length | 700 | 1,293 |
| Max Length | 1,7805 | 1,7805 |
| N50 | 2,382 | 2,541 |
| N90 | 511 | 827 |
| Total Nucleotides | 519,441,999 | 483,078,066 |

**Table S3. Statistics of unigene annotation rate.**

|  | **Number of Genes** | **Percentage (%)** |
| --- | --- | --- |
| Annotated in NR | 215,012 | 75.7 |
| Annotated in NT | 167,168 | 58.86 |
| Annotated in KO | 90,343 | 31.81 |
| Annotated in SwissProt | 163,015 | 57.4 |
| Annotated in PFAM | 152,313 | 53.63 |
| Annotated in GO | 154,112 | 54.26 |
| Annotated in KOG | 63,438 | 22.33 |
| Annotated in all Databases | 38,014 | 13.38 |
| Annotated in at least one Database | 231,334 | 81.45 |
| Total Unigenes | 283,998 | 100 |
