# Supplementary material for: Transcriptome Analysis of Oleoresin-Producing Tree Sindora Glabra and Characterization of Sesquiterpene Synthases
Source: Front Plant Sci. 2018 Nov 20;9:1619. doi: 10.3389/fpls.2018.01619 (PMC6256070; doi:10.3389/fpls.2018.01619)
Supplement: File S11 — Multiple sequence alignment of S. glabra TPS genes with homologous genes from other plants. [file Data_Sheet_1.PDF]

CLUSTAL O(1.2.4) multiple sequence alignment

RRX$_8$W

```
AGW18155.1      ----------------MARSTIGYTPGVWGNQFLSVASGPLMKNKKEEIHQHLQNLKQQL     44
AGW18154.1      ----------------MGRPTANFSSSVWGNQFLSIASGPLLKNKEAEIHQHLQNLKEQV     44
SgSTPS1         ----------------MGRPTANFSPSVWGNQFLSIASGPLLKNKEAEIHQHLLNLKEQV     44
AGW18156.1      MATEVSEHVALSSIQNADRPLVKYVPSIWGDFFLQYASEFMEVD--DNMKQKVGVLKEEV     58
APO20772.1      MSAQVLATVSSS-TEQTVRPIAGFHPNLWGDYFLTLASDCKTND--TTHQEEYEALKQEV     57
AIO10965.1      MSVPVSATISQNAKPKVIRKTANFQPSIWGDQFVTYTSDDAITH--AHKEKQVEELKEEV     58
AGW18157.1      MSVAALAIATSTPSSDVPRRSANYHPSVWGDHFLKYASQPLEVD--EKMEDRIGTLKETV     58
SgSTPS2         MSVAALAIPTSTPSSDVPRRSANYHPSVWGDHFLKYASQPLEVD--ERMEDHIGTLKETV     58
                                  *    :  .:**: *:  :*     .     ...   **: :

AGW18155.1      GRQLK-SVKEP-CEKLNLIDTMQRLGVSYHFQSEIEESLKHLHKNPPSSWNAKDINSHLL     102
AGW18154.1      RKQLKNGVEEP-SEKLNMIDTIQRLGVSYHFETEIVESLQQLHKNPPSSWDAEDVDAHLL     103
SgSTPS1         RKQLKNGVEEP-SEKLNLIDTIQRLGVSHHFEREIEESLKHLHKNPPSSWNAEDIDAHLL     103
AGW18156.1      RRMLVSSVNHNFSRKLDFIDSIQRLGVSYHFQHEIDEALKQIHDSFTNNAIITPSDHDLH     118
APO20772.1      RSMITATADTP-AQKLQLVDAVQRLGVGYHFEQEIEDALENIFHNSFDNN----DDVDLH     112
AIO10965.1      RRELMTRVDQP-SKQLKFIDAIQRLGVAYHFDREIEEALQHIYDDRLDNG-----DEDLY     112
AGW18157.1      RKMLVPATDKP-LTKVRLIDSIQRLGVDYHFESEIDEVLCQIQNNYVKDGIIT-LNEDLH     116
SgSTPS2         KKMLVPATDKP-LTKVKLIDSIQRLGVYYHFESEIDEVLCHIQKNYVKNGIIT-LDEDLH     116
                   :   ..     :: ::*::***** :**: ** : * ::  ..  ..      : .*
```

EDXXD

```
AGW18155.1      GTALWFRLLRQQGYYVSCDIFNKFKDDKGDFKTILIDDVEGMLALYEAAHLGIRGEEILD     162
AGW18154.1      SISLWFRLLRQQGYYVSCDVFNKFKDDKGVFKTALIDDVEGMLALYEAAYLGIRGEEILD     163
SgSTPS1         SISLWFRLLRQQGYYVSCDVFDKFKDDKGMFKTTLIDDVEGMLALYEATYLGIRGEEILD     163
AGW18156.1      SIALLFRLLRQQGYHVSSGIFIQYKDQNGNFNEKLRNDVRGMLSLYEAAQLRIDGDDILA     178
APO20772.1      TVSLRFRLLRQQGFKVSCDVFEKFKDDEGKFKASLVKEVQGILSLYEAGHLAIRGEDILD     172
AIO10965.1      NVSLWFRLLRQEGYNVSSDILYKFKNDEGNFKESLIGDVQGMLGLYEATHLRIHGEDVLD     172
AGW18157.1      SLALLFRLLRQQGYHVSPDVFNKFKDEQGKISETIANDVEGMLSLYEAAHLRIHGEDILD     176
SgSTPS2         SMSLLFRLLRQQGYHVSPGVFNKFKDEQGKISETIANDIEGMLSLYEAAHLRIQGEDILD     176
                  :* ******:*: ** .:: ::*:::* :.  :  ::.*:*.****  * * *:::*

AGW18155.1      QMLEFTMSYLKSRLK-GMT-PYLQERANRALHCPIHKGMLRIETRYYIPIYSKKDSRNDL     220
AGW18154.1      QVLEFTVFHLKSRLE-GMT-PYLQERVDRALYCPINKGLPRIETRYFISTYSKKDSRNDL     221
SgSTPS1         QVLEFTMFHLKSRLE-GMT-PYLRERADRALYCPINKGLPRIETRYFISMYSKKDSRNDL     221
AGW18156.1      EALDFTSTQLKLLSS-QLG-PSLVTEVEHSLRLPLHKTLQRIEARHYMSFYQDDPSHNEI     236
APO20772.1      EAIAFTRTHLQSMVSHDVRPNNLAEQINHALDCPLRRALPRVETRFFLSVYPRDDKHDKT     232
AIO10965.1      EALAFTTIHLESMATSRAS-DPLMAQVIHALKQPIRKGLPRLEAGRFISIYQEDGSHNEA     231
AGW18157.1      EALDFTSTHLKFLTT-QLS-DSHAGNVIRSLKRPLRMRLPRLEARHYFFTYQEDPSHIET     234
SgSTPS2         EALDFTSTHLKSLTT-QLS-GSLAGEVIRSLKRPLHRRLPRLEAWNYFSTYQEDPSHDKT     234
                : : **   *:              .  ::*  *:.  : *:*:  ::  *  . .: .

AGW18155.1      LLEFAILDFNILQQQYQKELSYITKWYKKLDFVSKVPYTRDRIVEGYFWPLATYFEKQCS     280
```

```
AGW18154.1      LLEFAMLDFNILQQQYQKELSHLTEWYKKLDFVSKVPYTRDRIVEGYFWPLGAYFENQYS      281
SgSTPS1         LLEFAMLDFNILQQQYQKELSHLSEWYKKLDFVSKVPYTRDRIVEGYFWPLGAYFENQYN      281
AGW18156.1      LLTFAKLDFDMLQKLHQNEIGNITKWWKKSDCARRVPYGRDRLVESYFWPLSISHEPQYS      296
AP020772.1      LLKFAKLDFNLVQRIHQKELSAITRWWKDLDFTTKLPYARDRIVELYFWIVGTYFEPKYT      292
AI010965.1      LLKLAKLDFNLLQSLHRKELSEIARWWKDLDFARKLPYVRDRVVECFFWILGVYFEPQYS      291
AGW18157.1      LLAFAKLDFNGLQKLHQREIGNLSKWWKDLDFATKLPFARNRLVEAYFWILGVYFEPCYS      294
SgSTPS2         LLTFAKLDFNRLQKLHQKEVGKLSKWWKDLDFATKLPFARNRLVEAYFWILGVYFEPCYS      294
                ** :* ***: :*   ::.*:.  ::.*:*. * .  ::*: *:*:** :** :.   .*   .

                                DDXXD
AGW18155.1      RGRRILGKMIGVFTCLDDTYDNFGTVDELNVLTEAIMRWDINLVASLPECMKVVFETTLD      340
AGW18154.1      KGRIIVSKLISVLTALDDTYDAYGTVDELKLFTEAIKRWDINMVASLPECMKVVFQAILD      341
SgSTPS1         KGRIIVSKLILVLTALDDTYDAYGTVDELKLFTEAIKRWDINMVASLPECMKVVFQAILD      341
AGW18156.1      IARSITGKLIAVLALLDDTYDAYGTVQELELFTEAVRRWDASLINFHPEGMKAVFGIITE      356
AP020772.1      LARKIMTKTIYTASIIDDTFDAYGFFEELKLFAEAVQRWDIGAMDILPEYMKVLYKALLD      352
AI010965.1      LARRILTKVISMTSVIDDIYDAYGTFEELELFTEAIERWDINFLDQLPEYMKFVYQALLD      351
AGW18157.1      LARRIMTKVISLTSIIDDIYDVYGTLEELQLFTEAIDKWDISCMDFLPEYMKLIYQPLLD      354
SgSTPS2         LARQILTKVISLTSVVDDIYDVYGTLEELQLLTEAIDRWDISCMDILPEYMKLIYQALLD      354
                 .* *  * *   : :** :* :* .:**::::**: :** .  :   ** ** ::     :

AGW18155.1      FLIEIELLTEEDGIS-FVVEYVKQGIQGLAKGYMVEAEWRAKGYIPTYDEYIENGIWTAG      399
AGW18154.1      LLSEMELLTEEDGIS-SFVEYVKPALQDLAKSYLLEAEWRDKSYIPTYEEYMANGVFSCG      400
SgSTPS1         LLDEMELLTEADGIS-CFVEYVKPALQDLAKSYLLEAEWRDKGYIPTYEEYIANGVFSCG      400
AGW18156.1      LCNEIESVIANEGKLNFIIEHVKHAIYNLAQAYLTETKWGNEGYIPTYSEYKSNGVATST      416
AP020772.1      TFNEIEQDLAKEGRS-SYLPYGKEKMQELVQMYFVQAKWFSEGYVPTWDEYYPVGLVSCG      411
AI010965.1      VYAEIEEEMSKQARA-YRVHYAKEAMKNLVQVYFVEAKWFNEGYIPPMEEYMPVALLSCG      410
AGW18157.1      VYDEIERETAKEGRA-FCVNYGKEQMRKVVRAYLAEAKWFHNNYTPTLEEYMEVAQVSSA      413
SgSTPS2         VYDEIERQAAKEGRA-FCVNYGKEEMRRLVRAYLAEAKWFHNNYTPAFEEYMEVAQVSSA      413
                   *:*      :.      : : *  :  :.: *: :::*  :.* *  .**   .   :.

                                                          NSE/DTE
AGW18155.1      YPALEITSLLALGNIATKEVFDWISSMPKIVRASGIVGRIGNDLGSHKREKNIGHVATSV      459
AGW18154.1      YPAVETTSLLGLGKTATKEVFDWISNVPKIVRASSIMCRLTDDLASHKFEQNREHVGSAI      460
SgSTPS1         YPAVEMASLLGLGKIATKEVFDWISNVPKIVRASSIMCRLTDDLASHKFEQNREHVGSAI      460
AGW18156.1      YPL-EIISFVSLTTLATEEVLNWISSDPEILKATSIIGRLLDDMASHKFEQERVHVASSV      475
AP020772.1      YFMLATNSFLGMCDVANKEAFEWISKDPKISTASSVICRLRNDIVSQQFEQKRGHIASGV      471
AI010965.1      YPMLITASFVGMGEVVTKEAFDWVFNNPKIVKASSIINRLMDDIVSHQFEQERGHVASGV      470
AGW18157.1      YSMLTTVSFIGVGSIATEEAFKWVTKDPKIVKASLIICRLMDDIVSSKFEQERGHVVSAL      473
SgSTPS2         YRMLTTVSFIGMGSIATEEAFKWITKNPKIVKASLVICRLMDDIVSGKFEQERGHVVSAL      473
                *      *::.:   ..:*.:.*: . *:*  *: :: *: :*: * : *::   *: :.:

AGW18155.1      ECYMKQYGVPEDEAYKLLLKEMENAWKDLNEEYMKPSS-IPKVVLDRVRNYMRANEFYYD      518
AGW18154.1      ECCMRQYEVSEEEAYKILLKEIENAWKDLNEEYMKPNG-VPKVVLKCVLNFSRVIEFLYG      519
SgSTPS1         ECYMKQYGVSEEEAYKMLLKEIENGWKDLNEEYMKPNG-VPKVVLKCVLNFSRVIEFLYG      519
AGW18156.1      ECCMKQYGISEEEAYKVLHDDITHYWNVLNEESLKLMNVIPKAVLEFLVNLARVSEIAYE      535
AP020772.1      ECYIKQYGVSEEEVVTVFTEEVENAWKDMNEEFLKPTA-FPVALIERPFNIARVIEFLNK      530
```

```
AIO10965.1      ECYMKQYGASKQEVFDEFNKQVVNAWKDINEEFLRPTD-VPFPLLLRALNFARVMDVLYK      529
AGW18157.1      ECYMKQHGATEEETIVEFRRRVENAWKDINEACLQPFE-VAKPLLMRSLNLSRVISLLYT      532
SgSTPS2         ECYMKQNGATEEETIVEFCRRVENAWKDINEDCLQPFE-VPKPLLMRSLNLSRVIYLLYM      532
                ** ::*    ::*.    :    : : *: :**  ::     .    ::     *  *.  .

AGW18155.1      RFVDNYT-IGEGMKDDVAAVFLDPIDIDHNK    548
AGW18154.1      HFVDKYT-NAEMLKDQIASLFVDPIAIELNK    549
SgSTPS1         HFVDKYT-NAEMLKDHIASLFVDPIAIDVNK    549
AGW18156.1      KYQDGYT-KGEFLKKYVDAVIVNPIP-----    560
APO20772.1      KG-DWYTHSH-AIKDQIAAVLRDPVTI----    555
AIO10965.1      DK-DGYTHVGKTTKDRIASLLIDPVAI----    555
AGW18157.1      DD-DCYTRSAGNTKKNIEALLINPVA-----    557
SgSTPS2         DD-DSYTHSSGNTKKNIEALLINPVA-----    557
                   * **      *. : ::: :*:
```

CLUSTAL O(1.2.4) multiple sequence alignment

```
AGW18158.1         ------------------------------------------------------------ 0
SgDTPS1            MSSANFFTLCSNNAIGSLPSSMHPIFLSSPLSFSNCYPTSKSFNGVSLFKSRAIPVNSSF 60
XP_010277558.1     MPNTTAF--LRLLP--PVP---------VSRTYPSSDPG-AVLSGVCSFATENKRSGFGL 46
OMP05060.1         ---------------------------------------M-TSFFGNWPLRGKDKGENFDI 21
EOX94746.1         -MSSHSIHHPFLS-SSPIPY--------SSISFSNKHPP-IPSAGNLRLWGKDKGENFDI 49
XP_021281194.1     -MSSHSIHQPFLTGFSPIPY--------SSISFSDKHPP-IPFAGNLRLWGKDKGENFDI 50
XP_002302110.1     --------------------------------------MA-SFSIGVWLYGARGKQDNFHA 22
XP_002520733.1     -MSSQSLHFLST-------A--------SSFSTPNSRPL-LSSGG-GLFGVKDTRITFDV 42
XP_021612241.1     MSTAQSFHLFSTVPATTRSS--------SSFSAIRHRPL-LFS-GVWLHGAKDKRGNVDI 50
XP_012092234.1     -MSSQPFHLLSSS--SSSSS--------SSFSPIRLRPT-LFSGGVWLYGAKDKQVNFDF 48

AGW18158.1         ------------------------------------------------------MNYEIQ 6
SgDTPS1            -RIKCSATSR--LSAPPSPDISGDKSGLPPFARIEILPSRKV----GEASKVSVINYEIQ 113
XP_010277558.1     LRSRRNA--IPRISAHVYTEILQ--NGLPTAEWLETL-KNDRE---GEAPKVS-ISKEIV 97
OMP05060.1         -RPLCSAISKPR--TQEYAGVFQ--NGLPVIKWK-EIVDDDIEQGEEEALKVF-EWNTIK 74
EOX94746.1         -RPLCSAISKPR--TQEYAGVFQ--NGLPVIKWK-EIVDDDIEQG--EALKVF-ESNKIK 100
XP_021281194.1     -RPLCSAISKPR--TQEYAGVLQ--NGLPVIKWK-EIVDDDIEEV--EALKVF-ESNKIK 101
XP_002302110.1     -HSRCSAISKPR--TQGYADLFHQQNGLPLINWPHDVVEDDTEE---DAAKVS-VAKEID 75
XP_002520733.1     -RPRCSALSNPP--TQEYPDVFQRN-GVAVIKRR-EVVEDAIEE---QVTKIS-VSNEIL 93
XP_021612241.1     -RPRCSAISNSR--TQEYPEVFQKN-GAPVIKWH-EIVEDDIQE---KVSKVS-ISTEII 101
XP_012092234.1     -RPRCAAVSQSQTHTQEYDIVFQKNGGVPVIKWH-EIVEDDIEE---QVPKVK-ISKEIT 102
                                                                          *
```

```
                                                                              QXXDGGWG
AGW18158.1          MRVDAVKAMWESIEDGWLNISAYDTAWVALVEDINGSGSPQFPSCLQWIVENQLPDGSWG  66
SgDTPS1             MRVDAVKAMWESIEDGWLNISAYDTAWVALVEDINGNSSPQFPSCLQWIVDNQLPDGSWG  173
XP_010277558.1      ERVDSIKAMLSSMEDGEISISAYDTAWVSLVQDILGGGAPQFPSSLLWIVDNQLPDGSWG  157
OMP05060.1          ERIDTIKAMLGSMEDGEISSSAYDTAWVALIEDVNGSGTPQFPISLEWIANNQLPDGSWG  134
EOX94746.1          ERVGTIKSMLGSMEDGEISSSAYDTAWVALVEDVNGGGTPQFPSSLEWIANNQLPDGSWG  160
XP_021281194.1      ERVDTIKSMLGSMEDGEISSSAYDTAWAALVEDVNRGGTPQFPSSLEWIANNQLPDGSWG  161
XP_002302110.1      EHVKTIKAMLEMMEDGEISISAYDTAWVALVEDINGSGLPQFPSSLQWIANNQLPDGSWG  135
XP_002520733.1      KRVQNVKSMLDSMEDGEISISAYDTAWVALVEDINGSGAPQFPSSLQWIANNQLSDGSWG  153
XP_021612241.1      KRIQSIKSMLDSMEDGEISISAYDTAWVALVEDVNGSGAPQFPSSLEWIANNQLSDGSWG  161
XP_012092234.1      ERVDSIKKMLESMEDGEITISAYDTAWVALVEDVNGSGAPQFPSSLQWIANNQLPDGSWG  162
                     ::  :* *   :*** :. *******.:*::*:  ..  **** .* **.:*** *****

AGW18158.1          DRAVFLSYDRLLSTLACVVALRHWNVHPEKSKRGIEFFKENLERLAKEDPANMSVGFEMI  126
SgDTPS1             DRAVFLSYDRLISTLACVIALRYWNVHPEKSQRGIEFFKENLERLAEEDPANMPASFEMI  233
XP_010277558.1      DHRIFSAHDRIISTLACVIALKSWNICPRKCDKGVVFIRENMSRLESENPEHMSIGFEVA  217
OMP05060.1          DRRIFMAHDRLLNTLACVIALKKWDVHPDKCQKGVCFFNENISKLEKENAEHMPIGFEVA  194
EOX94746.1          DRQIFMAHDRLINTLACVIALKTWDIHPDKCEKGVSFFKENISKLENENAEHMPIGFEVA  220
XP_021281194.1      DRQIFMAHDRLINTLACVVALKTWDVHPDKCEKGVSFFKENISKLENENAEHMPIGFEVA  221
XP_002302110.1      DAEIFLAHDRLINTLACVVALKSWNLHQEKCEKGMLFFRDNLCKLEDENAEHMPIGFEVA  195
XP_002520733.1      DGDIFTAHDRILNTLACVVALKSWNIHPDKCERGMKYFKENLCKLEDENAEHMPIGFEVA  213
XP_021612241.1      DAEIFTAHDRILNTLACVIALKTWNIHPDKCEKGMKYFKENLCKLEDENAEHMPIGFEVA  221
XP_012092234.1      DADIFTAHDRIINTLACVIALKSWNLHPDKAAKGMKYFKDNLCKLEDENAEHMPIGFEVA  222
                    *  :* ::**::.*****:**: *::   *. :*: ::.:*: :* .*:  :*  .**:

AGW18158.1          FPSLIEMARDLNIEVPDPNTHPILKQIYAMKNEKLKRIPMEVVHKMPTSLLFSLEAMPGL  186
SgDTPS1             FPSLIEMARNFDIEVPDPSTHPIFKQIYAMKNEKLKRIPMEVMHKMPTSLLFSLEAMPGL  293
XP_010277558.1      FPSLIEIARKLHLQVPV--DSTVMQMISAKRNLKLTRIPKEMMHIVPTTLLHSLEGMPGL  275
OMP05060.1          FPSLLQLARSLNIEVAY--DSPVFQDIYERRSLKLTRIPKEIMHKVPTTLLHSLEGMPGL  252
EOX94746.1          FPSLLEIARSLNIEVPY--DSPVFQDIYAKRSLKLTRIPKEIMYYVPTTLLHSLEGMPGL  278
XP_021281194.1      FPSLLEIARSLNIEAPY--DSRVFQDIYAKRSLKLTRIPKEIMQNVPTTLLHSLEGMPGL  279
XP_002302110.1      FPSLLEIAKKLDIEVPY--DSPVLQEIYASRNLKLTRIPKDIMHNVPTTLLHSLEGMPGL  253
XP_002520733.1      FPSLLELAKNLDIEVPE--DSPVLKEIYASRNIKLTKIPKDIMHKVPTTLLHSLEGMPGL  271
XP_021612241.1      FPSLIELARKLDIEVPE--DSPVLQEIYASRNLKLKKIPKDIMHKVPTTLLHSLEGMLGL  279
XP_012092234.1      FPSLIELASKLDIEVPE--DSPVLQQIYASRNLKLKKIPKDIMHKVPTTLLHSLEGMPDL  280
                    ****:::* .:.::.         ::: *   :.  **.:** :::   :**:**.***.* .*

AGW18158.1          QWDKLLKLQSENGSFLSSPASTAFALMQTKDKNCLRYLNDVVQKFSGAVPNFYSIEFFEQ  246
SgDTPS1             QWDKLLKLQSENGAFLSSPASTAFAVMQTKDKNCLRYLNDVVQKFNGAVPAFYPIDFFEQ  353
XP_010277558.1      DWEKLLKLQSPDGSFLFSPSSTAFALMQTKDENCLKYLKRVVERFNGGVPNVYPVDLFEH  335
OMP05060.1          DWEKLLKLQCKDGSFLFSPSSTAFALMQTKDENCLSYLNKTVQRFNGGVPNVYPVDMFEH  312
EOX94746.1          DWEKLLKLQCQDGSFLFSPSSTAFALMQTKDENCLRYLNRTVQRFNGGVPNVYPVDLFEH  338
XP_021281194.1      DWEKLLKLQCQDGSFLFSPSSTAFALMQTKDENCHRYLNKTVQRFNGGVPNVYPVDLFEH  339
XP_002302110.1      EWKRLLKLQSQDGSFLFSPSSTAFALSQTKDKNCMEYLNKAVQRFEGGVPNVYPVDLFEH  313
```

```
XP_002520733.1      EWEKLLKLQCPDGSFLFSPSSTAFALMQTKNENCLAYLNKIVQRFNGGVPNVYPVDLFEH  331
XP_021612241.1      DWEKLLKLQSQDGSFLFSPSSTAYALMQTKDENCLSYLNKIVQRFKGGVPNVYPVDLFEH  339
XP_012092234.1      EWDKLLKLQSQDGSFLFSPSSTAYALMQTKDDNCLAYLNKIVQRFKGGVPNVYPVDLFEH  340
                    :*.:*****.  :*:** **:***:*:  ***:.**   **:   *::*.*.** .* :::**:

                                                                     DXDDTAM
AGW18158.1          SWAIDRLTRLGISRYFGEKIKESMNFFYKNWKNTGLGWNRYTCDVPDLDDTIMAFRLLRL  306
SgDTPS1             GWAVDRLTRLGISRYFGEKIKESMDFYHKNWKSTGLSWSRYTCEVPDLDDTIMALRLLRL  413
XP_010277558.1      IWAVDRLERLGISRYFQSEIKECLDYVYRYWTEDGICWARN-STVHDIDDTAMAFRLLRL  394
OMP05060.1          IWSVDRLQRLGISRYFQPEIKECLDYIYRYWTEDGICWARN-TRVHDIDDTAMGFRILRL  371
EOX94746.1          IWTVDRLQRLGISRYFQPEIKECLDYVYRYWTEDAISWARN-TRVQDIDDTAMGFRLLRL  397
XP_021281194.1      IWTVDRLQRLGISRYFQPEIKECLDYVYRYWTEDGISWARN-TRVQDIDDTAMGFRLLRL  398
XP_002302110.1      IWAVDRLQRLGISRYFESQIDECVNYIHRYWTEDGICWARN-SEVHDIDDTAMGFRVLRL  372
XP_002520733.1      IWAVDRVQRLGISRYFRKELKECIDYVARYWEEDGICWARN-SAVHDIDDTAMGFRLLRL  390
XP_021612241.1      IWAVDRLQRLGISRYFKEELKESINYVARYWREDGICWARN-SEVHDIDDTAMGFRMLRL  398
XP_012092234.1      IWAVDRLQRLGISRFFEQELKECINYVARYWREDGICWARN-SEVHDIDDTAMGFRMLRL  399
                     *::**: ******:*  ::.*.:::  : * .  .: * *     * *:*** *.:*:***

AGW18158.1          HGYDISCDVLKHFETDGEFFCMVGQSSEAVTAMFNLFRASQVSFPGEKIMEDAKRFSCEF  366
SgDTPS1             HGYDISPDVLKHFEKDGKFFCFAGQSSEAVTVMFNLFRGSQVSFPGEKIMEDAKRFSRDF  473
XP_010277558.1      HGHDVSPDAFRHFEKGGEFFCFAGQSNQAITGMFNLYRASQVLFPGEKILEEAKTFSSRF  454
OMP05060.1          HGYEVSADVFRHFEKGGEFFCFVGQSNQAITGIFNLFRASQVMFPGDKILEEAKRFSSNF  431
EOX94746.1          HGYEVSADVFRHFEKDGEFFCFVGQSNQAVTGIFNLFRASQVLFPGDKILEDAKRFSSKF  457
XP_021281194.1      HGYEVSADVFRHFEKGGEFFCFVGQSNQAVTGIFNLFRASQVMFPGDKILEDAKRFSSKF  458
XP_002302110.1      NGHHVSADVFKHFEKGGEFFCFAGQSTAAVTGMFNLYRASQLLFPGEKILEKAKEFSFKF  432
XP_002520733.1      YGHEVSSDVFKHFKKGDTFFCFAGQSTQAVTGMYNLYRASQVLFPGEKVLEEAKEYSSSF  450
XP_021612241.1      YGHEVSADVFKHFKKGDTFFCFAGQSTQAVTGMFNLYRASQVMFPGEKILEEAKEFSSSF  458
XP_012092234.1      FGHDVSPDVFKHFKKGDTFFCFAGQSTQAVTGMFNLYRASQVLFPGENILEEAKEFSSTF  459
                     *:.:* *.::**:...  ***:.***.  *:* ::**:*.**:  ***::::*.** :*  *

AGW18158.1          LTEKRAANQLGDKWVIAKDIAGEIGFSLDLPWYGILPRIETRFYLDQYGGANDVWIAKVL  426
SgDTPS1             LTEKRAANQLGDKWVMAKDIAGEVGFALDFPWYGILPRIETRFYLDQYGGANDVWISKGL  533
XP_010277558.1      LSEKQASNQLLDKWIITKDLPGEVRYALDIPWYASLPRLEARYYLEQYGGEDDVWIGKTL  514
OMP05060.1          LTEKQASGELFDKWIITKDLPGEVGFALKIPWYASLPRVETRFYIEQYGGEDDVWIGKTL  491
EOX94746.1          LTEKQAADELLDKWIITKDLPGEVGFALKIPWYASLPRVETRFYIEQYGGEDDVWIGKTL  517
XP_021281194.1      LTEKQAADELLDKWIITKDLPGEVGFALKIPWYASLPRVETRFYIEQYGGEDDVWIGKTL  518
XP_002302110.1      LREKQAANELLDKWLITKDLPGEVGFALEIPWHASLPRVESRFYIEQYGGEDDVWIGKTL  492
XP_002520733.1      LKEKQEANEVLDKWIITKDLPGEVKYALDIPWYASLPRVESRFYLEQYGGEDDVWIGKTL  510
XP_021612241.1      LKEKQAANEVLDKWIITKDLPGEVEYSLDVPWYANLPRVESRFYIEQYGGEDDVWIGKTL  518
XP_012092234.1      LREKQAANEVLDKWIITKDLPGEVEYALDMPWFANLPRVEARFYIEQYGGEDDVWIGKTL  519
                    * **: :.:: ***:::**: **: ::*..**.. ***:*:*:*::**** :****.* *

AGW18158.1          YRLLRVNNEIYLELGKLDYNNCQALHRTEWAAVQEWYSESGLDQFGLDRDRLLVLFFLAS  486
SgDTPS1             YRVPRVNNEIYVELAKLDYNNCQALHRTEWAALQEWYSESGLEQFGLDTDRLLVLFFSGI  593
XP_010277558.1      YRMPLVNNNVYLELAKLDFNNCQALHQHEWVNLQKWYTDCNLGEFGVNRGTLLQAYYVAA  574
```

```
OMP05060.1          YRMENVNNNVYLELAKLDYNNCQALHQKEWDSIQKWYSEMNLGDFGVTRRSLLLTYFIAA 551
EOX94746.1          YRMRYVNNNVYLELAKLDYNNCQALHQMEWNSIQKWYSEMNLGDFGVSRRSLLLTYFMAA 577
XP_021281194.1      YRMRYVNNNVYLELAKLDYNNCQALHQMEWNSIQKWYSEMNLGDFGVSRRSLLLTYFMAA 578
XP_002302110.1      YRMPYVNNNEYLQLARLDYNNCQALHRIEWANFQKWYEECNLRDFGISRKTLLYSYFLAA 552
XP_002520733.1      YRMPYVNNNEYLDLAKLDYNNCQALHRKEWDNFQKWYEECELGNFGVSRRELLLAYFVAA 570
XP_021612241.1      YRMPYVNNNEYLQLARLDYNSCQALHRIEWDNFQKWYEECSLGDFGVSKRELLFCYFLAA 578
XP_012092234.1      YRMPFVNNNEYLELAKIDYNSCQALHRVEWDNFQKWYEECNLGAFGVSKRELLYAYYMAA 579
                    **:  ***: *::*.::*:*.*****: **  .*:** :  *  **:    **  :: .

AGW18158.1          SSVFEPERARERLAWVKTSALMEAITSTYNHQ-----RLRSAFVHEFTNATATS------ 535
SgDTPS1             LKYF-------------------------------------------------------- 597
XP_010277558.1      ASIFEPERWTERLAWARTAVLVEAVSLYLEKE----DPQRGAFVHDFFSNIGGSSIFSSD 630
OMP05060.1          ASIFEPERSQERLAWAKTAFLVETIASAFENEMKPTDEQRKAFVQVFRSVIDAR------ 605
EOX94746.1          ASIFEPERSQERLAWAKTAFLVEAIACSFDNEMRPS-EQRKAFVQVFRSVVDAR------ 630
XP_021281194.1      ASIFEPERSQERLAWAKTAFLVEAIASSFDNEMRPS-EQRKAFVQVFRSVVDAR------ 631
XP_002302110.1      ASVFEPERSNERLAWAKTTILLEMIHSYFHEDDDNSGAQRRTFVHEFSTGISIN------ 606
XP_002520733.1      ASIFEPERSKERLAWAKTTTLLHTIESYFDAS-NSTYEQRTAFVHEFKNGVAS------- 622
XP_021612241.1      ASIFEPERSKERLAWAKTIVLLETIDSYFDEN-NSSIEQRRAFVQEFKNGAEA------- 630
XP_012092234.1      ASIFEPERSKERVAWAKTTILLRTIDAYFNDNSNNSVEERKDFVQQFKHGVGAP------ 633
                     . *

AGW18158.1          ------LRSSKVNERSPGLVNTLMKTLHDISLSTSTAHY----GTLQKMWKKWLLRWESE 585
SgDTPS1             ------------------------------------------------------------ 597
XP_010277558.1      NKNKLDKRRWGSKRTAERLVEALLGTLNRLSLDSLLAHGQDVLLHLRRAWATWLLISLDE 690
OMP05060.1          -FSHISGRKLNANRTIQKLTDILLRTLNHLSLDALVAHGRDISSSIRRAWEKWMMMWVEE 664
EOX94746.1          -FSHINGRKLDSNRRVQKLIDTLLRTLNHLSLDALVAHGRDISCSIRRAWEKWMLMWLEE 689
XP_021281194.1      -FSHINGRKLDSNRRVQKLIDTLLRTLNHLSLDALVAHGRDISCSIRRAWEKWMLMWLEE 690
XP_002302110.1      -----GRRSGTKK-TRKELVKMLLGTLNQLSFGALEVHGRDISHSLRHAWERWLISWELE 660
XP_002520733.1      -IPHLNARKLEVK-TNEELVRIAIGILNDVSLDTLLAHGKDISHDLRHAWEKWLLKWAEG 680
XP_021612241.1      -RGPVNGRTMEAK-TRQELVRIVLGTLNDVSLDALVAHGRDISHSLRHAWQNWLLKWEEE 688
XP_012092234.1      -AGLVNGRKLETK-TRQELVGIVLGTLNDVSLDALVAHGTDISHSLRHAWEKWLTNWEKE 691


AGW18158.1          GD---------DCEGGAELLANMININAGYFLSRKLQ-LNPEYQRLVQLTNQLCHRLQSL 635
SgDTPS1             ------------------------------------------------------------ 597
XP_010277558.1      AEEEEEEEDKQQTGREAELLVRTINLCAGRPLSEELL-CHPHYRRLVQLTNRVCHHLRRF 749
OMP05060.1          GD---------RQQGVAELVVQTINLSCGRCSLEELL-SHPKYQRLSNLTNSVCHQLSHY 714
EOX94746.1          GD---------RHQGVAELVVQTINLSSGRWSLEELL-SHPQYERLSSLTNTVCHQLCHY 739
XP_021281194.1      GD---------RHHGVAELVVQTINLSSGRWSLEELL-SHPQYERLSSLTNTVCHQLCHY 740
XP_002302110.1      GD---------RRRGEAELLVQTIHLTAGYKVSEELLVYHPQYEQLADLTNRICYQLGHY 711
XP_002520733.1      GE---------IHQGTGELLVKTITLTAGGSTP-----DHHKYAQLFQLTDKLCYQLAHY 726
XP_021612241.1      GD---------RHQGEAELIVKTINLAAGRWISEELLSCHSQYEKLFQLTNRICYQLGHY 739
XP_012092234.1      GD---------RHQGEAELIVKTINLTTGRWVSEELLNYSSKYERFFQLTNRICHQLGHH 742
```

```
AGW18158.1          QNSKEPASSNNSNK----TGLSDPEIESKMQELVQLVLLN--SSNGIDSNIKKTFLALTK 689
SgDTPS1             ------------------------------------------------------------ 597
XP_010277558.1      REWKVNERSSDDPNT---SSITTCEIESDMQELVQCVLR---SSDGIDPAIKQTFLAVAK 803
OMP05060.1          QKQKVQENGCYNADTD---NIRTQKIDSDMKQLVQLVLASSSSDDGINSDINQTFLTVAR 771
EOX94746.1          QKQKVHDNGCYNTDTD---NSRSQKIESDMQQLVQSVLQH--CSDGINPDIKHTFLTVAR 794
XP_021281194.1      QKQKVHDNGCYNTDTG---NSRSQKIESDMQQLVQSVLQH--CSDGINPDIKHTFLTVAR 795
XP_002302110.1      QKNKVHDNGSYSTITGSTDRITTPQIESDMQELMQLVIQK--TSDGIDPKIKQTFLQVAK 769
XP_002520733.1      RKNKVQGN----------KKSTTPEIESDMQQLVQLAIQN--SSDEIDSEIKQTFFMVAK 774
XP_021612241.1      RKNKVNDN----------KRSTTTEIETEMQELMQQVIQN--SSDGMDSNIKETFFTVAK 787
XP_012092234.1      SKNKVYDR----------ERSTTPEIESDMQELTQLVLQN--SSDGMDSNIKQTFFTVVK 790


AGW18158.1          TFYYAAYCDSKTIDTHIAKVLFERVN     715
SgDTPS1             --------------------------     597
XP_010277558.1      SYYYTAHCPPAMINLHIAKVLFESVH     829
OMP05060.1          SFYYAAHCDLDTITFHIAKVLFEKVR     797
EOX94746.1          SYYYAAHCDLETMTFHIAKVLFEKVR     820
XP_021281194.1      SYYYAAHCDLETITFHIAKVLFEKVR     821
XP_002302110.1      SFYYTAFCDPGTINYHIAKVLFETVA     795
XP_002520733.1      SFYYQAISDPGTLNYHIARVLFERVY     800
XP_021612241.1      SFYYTAICDPGTINYHISKVLFERVY     813
XP_012092234.1      SFYYTAVCDPGTINYHISKVLFEKVY     816

CLUSTAL O(1.2.4) multiple sequence alignment

SgDTPS2             ------------------------------------------MSISRPFILQN-SISPTR 17
SgDTPS3             ------------------------------------------------------------ 0
SgDTPS4             ------------------------------------------------------------ 0
XP_004488548.1      ------------------------------------------MSLSRFITPLSCISSTSD 18
XP_007149183.1      QNKAPHTTHALHFVRELYIYLPPL--WCLQQQHHSFHSL-DAMSLSRLITPLFCSSSTSA 57
KHN21375.1          -----------------------------------------------------MI-SFLT 6
XP_006585394.1      ----------MHFTL-LHTYTCPHFGVCNSSNTIFFHSLDDAMSLSHLTTPLCCT-SSTS 48


SgDTPS2             ASLKPGLNVRMKSNTATLLIEESKEKFQKMFKKVELSVSSYDTAWLALIPSPSSPDAPFF 77
SgDTPS3             ----------MKSNTATLLIEESKEKFQKMFKKVELSVSSYDTAWLALIPSPSSPDAPFF 50
SgDTPS4             ------------------------------------------------------------ 0
XP_004488548.1      SSMIT-STEKKNKNTTTLCFEDTKERVKNMFNKVELSISSYDTAFVAMIPSSTSSHAPLF 77
XP_007149183.1      DSLVAASYVKEKMESTSWCLADSKEKIRKMFNKVELSISSYDTAWVAMITSPASPHNPLF 117
KHN21375.1          DSLLTASCVKKKMNSTALCHEASKERIRKLFNKVELSVSSYDTAWVAMIPSPASPHTPFF 66
XP_006585394.1      DSLLTASCVKKKMNSTALCHEASKERIRKLFNKVELSVSSYDTAWVAMIPSPASPHTPFF 108
```

```
                              QXXDGGWG
SgDTPS2               PECLKWLLKNQLCDGSWGLPDRHPLLMKDTLLSTLACVLALKQWNVGEEHINRGLQFIES 137
SgDTPS3               PECLKWLLKNQLCDGSWGLPDRHPLLMKDTLLSTLACVLALKQWNVGEEHINRGLQFIES 110
SgDTPS4               ---------------------------MKDTLLSTLACVLALKQWNVGEEHINRGLQFIES 34
XP_004488548.1        PQCLNWLLDNQLLDGSWGLPGRHPLLTNDALLSTLACILALKQWGIGEDKMNKGLEFIES 137
XP_007149183.1        PQCVNWLLANQLLDGSWGLPDRHPLLMNDALLSTLACILALKQWGVGEDQINRGLEFIES 177
KHN21375.1            PQCLNWLLYNQLLDGSWGLPDRHPLLMNDALLSTLASILALKQWGVGEDQINRGLRFIQS 126
XP_006585394.1        PQCLNWLLYNQLLDGSWGLPDRHPLLMNDALLSTLASILALKQWGVGEDQINRGLRFIQS 168
                                                 :*:******.:******.:**:::*:**.**:*

SgDTPS2               NFASANDEKLQSPIGFDIIFPSLIENAQNLGIILPLQAKILEAVNQKRELELKRCYQSNS 197
SgDTPS3               NFASANDEKLQSPIGFDIIFPSLIENAQNLGIILPLQAKILEAVNQKRELELKRCYQSNS 170
SgDTPS4               NFASANDEKLQSPIGFDIIFPSLIENAQNLGIILPLQAKILEAVNQKRELELKRCYQSNS 94
XP_004488548.1        NFTSINDDKQHHPIGFHILFPSLIEYAQTLGISLPIRSTSLEAMIQRRDNELQRGFQSNS 197
XP_007149183.1        NITSIKDEKQHLPIGFGINFPSLIEYAQNLGINLPIGATLLDTMVQNREIELQRGTESNS 237
KHN21375.1            NITSINDENQHPPIGFGILFPSMIEYAQNLGINLPIGATSLEAMIQKREIELHRGSQSNS 186
XP_006585394.1        NITSINDENQHPPIGFGILFPSMIEYAQNLGINLPIGATSLEAMIQKREIELHRGSQSNS 228
                      *::* :*:: : **** * ***:** **.*** **: :. *::: *.*: **:*  :***

SgDTPS2               EGSRAYLAYISEGIGKSQDWQMVMKYQRKNGSLFNSPSTTAAAFTRLQNADCLKYLQMLL 257
SgDTPS3               EGSRAYLAYISEGIGKSQDWQMVMKYQRKNGSLFNSPSTTAAAFTRLQNADCLKYLQMLL 230
SgDTPS4               EGSRAYLAYISEGIGKSQDWQMVMKYQRKNGSLFNSPSTTAAAFTRLQNADCLKYLQMLL 154
XP_004488548.1        EGWREYLAYLSEGMLKSLDSNTIMKYQRKNGSLFNSPATTAAVFQHLKNADCLSYLQSVL 257
XP_007149183.1        EGWRAYQAYVSEGMLDSQDWKTIIKYQRKNGSLFNSPATTAAVFQRLKNAECLGYLQSVL 297
KHN21375.1            DGRRAYLAYVSEGMLESQDWKSIMKYQRKNGSLFNSPATTAAVFQCHKNAECLGYLQSVL 246
XP_006585394.1        DGRRAYLAYVSEGMLESQDWKSIMKYQRKNGSLFNSPATTAAVFQCHKNAECLGYLQSVL 288
                      :* * * **:***: .* * : ::*************:****.*   :**:** *** :*

SgDTPS2               EKFGNAVPTIYPLDTYARLCMIDSLQRLGIDYHFRKEIQSVLDDTYRHWLHGEEDIFLDP 317
SgDTPS3               EKFGNAVPTIYPLDTYARLCMIDSLQRLGIDYHFRKEIQSVLDDTYRHWLHGEEDIFLDP 290
SgDTPS4               EKFGNAVPTIYPLDTYARLCMIDSLQRLGIDYHFRKEIQSVLDDTYRHWLHGEEDIFLDP 214
XP_004488548.1        EKFGNAVPTVYPLDIYARLYMIDSLERMGINHHFKEEIRSVLDETYRYWLQGEENIFLDP 317
XP_007149183.1        EKFGNAVPTTHPLDIYARLCMIDSLERLGINHHFKEEIRSVLDDTYRFWVQGVEDIFLDP 357
KHN21375.1            EKFENAVPTTYPLDIYARLCMIDSLERLGINHHFKEEIRSVLDEIFRYWMQGVEDIFLDP 306
XP_006585394.1        EKFENAVPTTYPLDIYARLCMIDSLERLGINHHFKEEIRSVLDEIFRYWMQGVEDIFLDP 348
                      *** ***** :*** **** *****:*:**::**::**:****: :*.*::* *:*****

SgDTPS2               TTCAIAFRLLRLNGYDVSSEIFDQYTEDKFSNSLKGYLKDVGAILEIYRASQIVTSADES 377
SgDTPS3               TTCAIAFRLLRLNGYDVSSEIFDQYTEDKFSNSLKGYLKDVGAILEIYRASQIVTSADES 350
SgDTPS4               TTCAIAFRLLRLNGYDVSSEIFDQYTEDKFSNSLKGYLKDVGAILEIYRASQIVTSADES 274
XP_004488548.1        TTCAMAFRLLRLNGYDVSSDPFYQYSEDKFSNSLKGYLKDVSAVLELYRASQFIIHPDES 377
XP_007149183.1        TTCAMAFRILRLNGYDVSSDPFYQYSEDKFGDSLKGYLKDVGAVIELYRASQTIIHPDES 417
KHN21375.1            TTCAMAFRMLRLNGYDVSSDPFYQYSEDKFAESLKGYLKDVGAVIELYRASQAIIHPDES 366
XP_006585394.1        TTCAMAFRMLRLNGYDVSSDPFYQYSEDKFAESLKGYLKDVGAVIELYRASQAIIHPDES 408
                      ****:***:**********: * **:****.:*********.*::*:***** :   ***
```

```
SgDTPS2             ILVKQNDWTGHLLKQDT-TYPGYVDRFRNYIEHEVKDALKFPSYANLERLLNRRSIDHYN 436
SgDTPS3             ILVKQNDWTGHLLKQDT-TYPGYVDRFRNYIEHEVKDALKFPSYANLERLLNRRSIDHYN 409
SgDTPS4             ILVKQNDWTGHLLKQDT-TYPGYVDRFRNYIEHEVKDALKFPSYANLERLLNRRSIDHYN 333
XP_004488548.1      ILVKQRSWTRRLLKQDSSPYQLYADKLRIYVDNEVNDILNFPHHANLERLLNRRSVEHYN 437
XP_007149183.1      ILVRQSLWTKQLLKQESSPYRLYADKLRSYVDQEVKDVLSFPHHANLERLLNRRSMEYYN 477
KHN21375.1          ILVRQSLWTKHLLKQESSPYRLYADKLRSYVDLEIKDVLNFPYHANLERLLNRRSMEHYN 426
XP_006585394.1      ILVRQSLWTKHLLKQESSPYRLYADKLRSYVDLEIKDVLNFPYHANLERLLNRRSMEHYN 468
                    ***:*  ** :****::  *  *.*::* *:: *::* *.** :***********:::**

SgDTPS2             VDNTRILKTSYRSYNLGNQEILNLAVEDFNNCQSIHREELKILGRWVVESRLDKLKFARQ 496
SgDTPS3             VDNTRILKTSYRSYNLGNQEILNLAVEDFNNCQSIHREELKILGRWVVESRLDKLKFARQ 469
SgDTPS4             VDNTRILKTSYRSYNLGNQEILNLAVEDFNNCQSIHREELKILGRWVVESRLDKLKFARQ 393
XP_004488548.1      ADETRILKTSYRSCNLANQEILKLAVEDFNLCQSIHNKELKQLARWIVTSRLDKLEFARQ 497
XP_007149183.1      VEETRILKSSYRSCNLANQEILKLAAEDFNICQSIHIEELKQLSRWVVESRLDKLTFARQ 537
KHN21375.1          TVETRILKASYRSCNLANQEILKLAVEDFNICQAIHIEELKQLSRWVVERRLDTLKFARQ 486
XP_006585394.1      TVETRILKASYRSCNLANQEILKLAVEDFNICQAIHIEELKQLSRWVVERRLDTLKFARQ 528
                    . :*****:**** **.*****:**.**** **:** :*** *.**:*  ***.* ****

                                                             DDXXD
SgDTPS2             KLAYCYFSSSATLYLPELSDARISWAKNGVLTTVVDDFYDVGGSEEELANLIELFEKWDV 556
SgDTPS3             KLAYCYFSSSATLYLPELSDARISWAKNGVLTTVVDDFYDVGGSEEELANLIELFEKWDV 529
SgDTPS4             KLAYCYFSSSATLYLPELSDARISWAKNGVLTTVVDDFYDVGGSEEELANLIELFEKWDV 453
XP_004488548.1      KLAYCYFSSAATLFSPELSDARISWAKNGVLTTVVDDFFDVGSSEEEQVNLIQLVEKWDV 557
XP_007149183.1      KLAYCYFSGAATLFSPELSDARISWAKNGVLTTVVDDFYDVGGSEKELVDLIQLVEKWDV 597
KHN21375.1          KLAYCYFSCAATIFSPELSDARISWAKSGVLTTVVDDFFDVGGSEEEHVNLIQLVEKWDV 546
XP_006585394.1      KLAYCYFSCAATIFSPELSDARISWAKSGVLTTVVDDFFDVGGSEEEHVNLIQLVEKWDV 588
                    ******** :**:: ************.**********:***.**:* .:**:*.*****

SgDTPS2             DVNTECCSEQVAIIFSAIRSTICEIEEKAFKWQGRSVKNHVINIWLDLIQSMMKEAQWLK 616
SgDTPS3             DVNTECCSEQVAIIFSAIRSTICEIEEKAFKWQGRSVKNHVINIWLDLIQSMMKEAQWLK 589
SgDTPS4             DVNTECCSEQVAIIFSAIRSTICEIEEKAFKWQGRSVKNHVINIWLDLIQSMMKEAQWLK 513
XP_004488548.1      DVDTVCCSEAVKIIFSAVRSTICEIGEKSVERQGRNVKDNVIKIWLNLMKSMYTEAEWLR 617
XP_007149183.1      DINTVSCSETVKIIFSAIHSTVCEIGERSVKRQGRNVKNNVIKIWLDLIQSMYKEAEWLR 657
KHN21375.1          DINTVCCSETVKIIFSAIHSTVCEIGEKSVKQQGRNVKNNVIKIWLNLVQSMFREAEWLR 606
XP_006585394.1      DINTVCCSETVKIIFSAIHSTVCEIGEKSVKQQGRNVKNNVIKIWLNLVQSMFREAEWLR 648
                    *::* .*** * *****::**:*** *::.: ***.**::**:***:*::**  **:**:

SgDTPS2             SKSVPTIDEYMANAYISFALGPIVLPTLYLVGPKLPTTLAENPEFNCLYELMSTGGRLLN 676
SgDTPS3             SKSVPTIDEYMANAYISFALGPIVLPTLYLVGPKLPTTLAENPEFNCLYELMSTGGRLLN 649
SgDTPS4             SKSVPTIDEYMANAYISFALGPIVLPTLYLVGPKLPTTLAENPEFNCLYELMSTGGRLLN 573
XP_004488548.1      TKTIPTIDDYMQNAYVSFALGPIVLPALYLVEPKLSDDVAENQELDCLFKTMSTCGRLLN 677
XP_007149183.1      TKTVPTIDEYMENAYVSFALGPIVLPALYLVGPKLSDEDAESHELNHLYKLMSTCGRVLN 717
KHN21375.1          TKTVPTIGDYMENAYISFALGPIVLPALYLVGPKLSDEVTENHELNYLYKLMSTCGRLLN 666
XP_006585394.1      TKTVPTIGDYMENAYISFALGPIVLPALYLVGPKLSDEVTENHELNYLYKLMSTCGRLLN 708
```

```
                    :*::***.:** ***:**********:**** ***     :*.  *::  *::  ***  **:**
                   NSE/DTE
SgDTPS2             DIHSFKRESEEGKLNAVGLRLVHGNGAIAAEDVIKEMKGVAEDKRRELLRLVLQ-KGSSI  735
SgDTPS3             DIHSFKRESEEGKLNAVGLRLVHGNGAIAAEDVIKEMKGVAEDKRRELLRLVLQ-KGSSI  708
SgDTPS4             DIHSFKRESEEGKLNAVGLRLVHGNGAIAAEDVIKEMKGVAEDKRRELLRLVLQ-KGSSI  632
XP_004488548.1      DIHSFKRESEEGKLNAVSLHMVHGNGVVTAEDAVNKMKGVIEDKRRELLRLVLQEKGSLV  737
XP_007149183.1      DIHSFKRESEEGKLNALGLLIAHGNGVFTAEDAIEKLKGTAEEKRTELLRLILHEKGSVV  777
KHN21375.1          DIHSFKRESEEGKLNVLALRIAHGNGVITAEDATEEMKGIAEEKRRELLRLILQEKGSVV  726
XP_006585394.1      DIHSFKRESEEGKLNVLALRIAHGNGVITAEDATEEMKGIAEEKRRELLRLILQEKGSVV  768
                    ***************.:.* :.****..:***.  :::**  *:** *****:*: *** :

SgDTPS2             PRACKDLFWKMARVLNLFYIKDDGFTSNEMISIVKALLEEPVVLDDRGAVADVQ------  789
SgDTPS3             PRACKDLFWKMARVLNLFYIKDDGFTSNEMISIVKALLEEPVVLDDRGAVADVQ------  762
SgDTPS4             PRACKDLFWKMARVLNLFYIKDDGFTSNEMISIVKALLEEPVVLDDRGAVADVQ------  686
XP_004488548.1      PRDCKDLFWKMMKVLNLFYIKDDGFTSNEMHSTVNAVIKEPIILNDLLVDSKQH------  791
XP_007149183.1      PRDCKDLFWKMIKVLHLFYLKDDGFTSNEMYSSVKAVIKDPVIFDELLARQQ-NLNYVEA  836
KHN21375.1          PRECKDLFWKMIKVLHLFYMKDDGFTSHEMHSSVNAVLKKPVILNELLVHSQQNLSPVKA  786
XP_006585394.1      PRECKDLFWKMIKVLHLFYMKDDGFTSHEMHSSVNAVLKKPVILNELLVHSQQNLSPVKA  828
                    ** ******** :**:***:*******:** * *:*:::.*:::::  .   .  :

SgDTPS2             --------   789
SgDTPS3             --------   762
SgDTPS4             --------   686
XP_004488548.1      --------   791
XP_007149183.1      SNVMPQSS   844
KHN21375.1          TNDLML--   792
XP_006585394.1      TNDLML--   834
```
